# Time course of energy expenditure in persistent critical illness: a prospective multicentre study

**DOI:** 10.1186/s13054-026-06102-w

**Published:** 2026-05-27

**Authors:** Timo Oosterveld, Michelle C. Paulus, Benjamin Hess, Henrike Häbel, Åsa Johansson, Nicole Mürner, Annika Reintam Blaser, Kate Fetterplace, Emma J. Ridley, Oana A. Tatucu-Babet, Arthur R. H. van Zanten, Michael Wanecek, Kym Wittholz, Adam Deane, Olav Rooyackers, Martin Sundström Rehal

**Affiliations:** 1 Department of Perioperative Medicine and Intensive Care (PMI), Karolinska University Hospital Huddinge, Stockholm, Sweden; 2 Division of Anesthesia and Intensive Care, Department of Clinical Science, Intervention and Technology (CLINTEC), Karolinska Institutet, Stockholm, Sweden; 3 Department of Intensive Care Medicine, Gelderse Vallei Hospital, Ede, The Netherlands; 4 Division of Human Nutrition and Health, Wageningen University & Research, Wageningen, The Netherlands; 5 Department of Intensive Care Medicine, Lucerne Cantonal Hospital, Lucerne, Switzerland; 6 Department of Learning, Informatics, Management and Ethics, Karolinska Institutet, Stockholm, Sweden; 7 Department of Anaesthesiology and Intensive Care, Orebro University Hospital, Örebro, Sweden; 8 Department of Anaesthesiology and Intensive Care, University of Tartu, Tartu, Estonia; 9 Australian and New Zealand Intensive Care Research Centre, School of Public Health and Preventive Medicine, Department of Epidemiology and Preventative Medicine, Monash University, Melbourne, Australia; 10 Nutrition Department, The Alfred Hospital, Melbourne, Australia; 11 Intensive Care Unit, Royal Melbourne Hospital, Melbourne, Australia; 12 Intensive Care Unit, Capio Saint Göran’s Hospital, Stockholm, Sweden; 13 Department of Critical Care, Melbourne Medical School, University of Melbourne, Melbourne, Australia

**Keywords:** Critical illness, Metabolism, Indirect calorimetry, Nutrition

## Abstract

**Background:**

Metabolic alterations are a fundamental part of critical illness, but changes during prolonged ICU stay are inadequately understood. This study aimed to describe longitudinal trends in energy expenditure and substrate utilisation in persistent critical illness, defined as an ICU stay of ≥ 10 days.

**Methods:**

This prospective, observational, multicentre study was conducted from 2022 to 2024 at five European and two Australian ICUs. The primary outcome was the change in energy expenditure over time. Adult patients with ≥ 1 indirect calorimetry and length of stay ≥ 10 days were included. Clinical parameters, markers of inflammation and protein catabolism were collected at each measurement. Longitudinal trends were analysed using mixed-effects models with restricted cubic splines. Latent class analysis was performed with identical covariates.

**Results:**

433 patients with 1194 measurements were included. The mean age was 56 years, and 70% were male. An initial increase in energy expenditure, peaking around day 10, and subsequent decline were found (*p* < 0.001), remaining significant after adjustment for sex, age, CRP, FiO2, presence of fever, BMI, renal replacement therapy, administered protein as fixed effects and patient and site as random effects (*p* = 0.001, conditional R² = 0.76). The association between the respiratory quotient and time was non-significant (*p* = 0.067). The urea: creatinine ratio increased over the first 10 days (*p* < 0.001). Latent class analysis identified three trajectories of energy expenditure: hypo-, normo-, and hypermetabolism (entropy 0.63).

**Conclusions:**

Mean energy expenditure follows a biphasic pattern during prolonged ICU stay, with the inflection point coinciding with the empirical onset of persistent critical illness. Further research is required to validate potential metabolic subgroups and explore their biological correlates in this population.

**Trial registration:**

The study, including the statistical analysis plan, was prospectively registered at clinicaltrials.gov (NCT05124860, registered 2021-11-15).

**Supplementary Information:**

The online version contains supplementary material available at 10.1186/s13054-026-06102-w.

## Introduction

Alterations in metabolism are central to the physiological changes in critically ill patients. Regardless of the primary insult, a similar pattern of insulin resistance, mobilisation of endogenous energy reserves and protein catabolism is generally observed early in intensive care unit (ICU) stay [1]. These changes typically resolve after the acute phase or transition into persistent critical illness [2–4]. Attempts to delineate this condition have focused on markers of inflammation, immune dysfunction and protein catabolism [5–7]. Another approach has been to identify the time point when admission severity scores lose discriminative power, suggesting that factors outside the circumstances of ICU admission drive outcome. Multiple studies have observed this transition occurring around day 10 in ICU [4, 8].

Patients with persistent critical illness have worse outcomes and consume a disproportionate amount of ICU resources [4, 8]. Understanding the underlying mechanisms and metabolic changes is essential to advancing research and clinical care [9]. Most works describing metabolic changes in ICU patients have focused on the acute phase, limiting extrapolations to patients with prolonged ICU stays.

Energy expenditure (EE) is a fundamental property of metabolism that can be readily measured at the bedside [3]. It also has direct implications for nutritional management in the ICU, especially in prolonged ICU stay. This study aimed to characterise the time course of energy expenditure and indices of substrate utilisation, protein catabolism, and inflammation in a cohort of patients with persistent critical illness, defined as an ICU stay ≥ 10 days. We hypothesised that energy expenditure and the respiratory quotient would change between the acute and post-acute phase in ICU.

## Materials and methods

### Setting and Participants

This prospective, observational multicentre study was conducted at seven ICUs in Sweden, the Netherlands, Switzerland and Australia (described in Supplemental Table 1), pre-registered at ClinicalTrials.gov (NCT05124860, registered 2021-11-15) with primary ethical approval from the Swedish Ethical Review Authority (2021–02750). Local ethical approval was obtained at all participating sites with a waiver of informed consent, except one centre where informed consent was obtained (Supplemental Table 1). Patients ≥ 18 years with at least one indirect calorimetry measurement were eligible for enrolment and included in the primary analysis if they remained in the ICU 10 days or more. Exclusion criteria were pregnancy, prior enrolment, and burns > 20% body surface area.

### Outcomes and subgroup analyses

The primary outcome was the change in energy expenditure over time. Secondary outcomes included changes in the respiratory quotient (RQ), oxygen consumption (VO_2_), and carbon dioxide production (VCO_2_). An analysis of patients with < 10 days ICU stay was planned as a secondary outcome, but was omitted due to variable enrolment at participating sites. As an exploratory outcome, latent class analysis (LCA) was performed to identify subgroups based on metabolic rate and examine their characteristics. Pre-planned sub-analyses included the sequential organ failure assessment score (SOFA) in the model for European patients, since this variable was not routinely collected at the two Australian sites. European and Australian sites were also compared for systematic differences. As patients with longer stay can skew the results of regression models, patients in the top length of stay quartile were compared to the full cohort. A sensitivity analysis was performed to determine the effect of outlier values for RQ on model prediction.

### Data collection and management

Indirect calorimetry was performed per local routine with recommendations for measurement conditions and calibrations provided in the protocol supplement (Supplemental Table 2). Data were collected using REDCap and securely stored at Karolinska Institutet [10]. Quality control included site monitoring after initial enrolment and continuous consistency checks. Final validation of energy expenditure and RQ values against VO_2_ and VCO_2_ was performed on the complete data set by comparing measured energy expenditure with calculated energy expenditure using VO_2_ and VCO_2_. For data normalized to body weight, the actual admission body weight was used when body mass index (BMI) ≤ 25. Otherwise, adjusted body weight was calculated as the ideal body weight plus 25% of the difference between actual and ideal weight. The complete variable list, including calculations, is provided in Supplemental Table 3.

### Missing data

The extent and pattern of the missingness were explored. As per the pre-specified analysis plan, missingness < 3% was ignored. Variables with > 30% missingness were not included in any models, with the exception of the SOFA score, as described above. CRP was linearly interpolated but not extrapolated. Albumin was not imputed.

### Statistical analysis

The statistical plan was made publicly available prior to data analysis at clinicaltrials.gov (NCT05124860, registered 2021-11-15).

Based on Swedish registry data where 5% of ICU patients remain in the ICU for more than 10 days, a target sample size of at least 200 patients with repeated measurements was chosen a priori [11].

Numerical variables were summarised as means with standard deviations (SD) or medians and interquartile ranges (IQR) as appropriate. Categorical variables were presented as frequencies (proportions). Due to right-skewed distributions, energy expenditure, RQ, VO_2_, and VCO_2_ were log-transformed. Time trends were described using rolling medians (IQR) and locally estimated scatterplot smoothing (LOESS).

Associations between energy expenditure, RQ, VO_2_, and VCO_2_, and explanatory variables were assessed using linear mixed-effects models (LMMs) with patient and site as random effects. The model allowed a random intercept and slope over time for patients, but only a random intercept for the site. An unstructured covariance matrix was used. Covariate selection followed an automated backwards elimination strategy. The final model for energy expenditure included time, age, sex, fraction of inspired oxygen (FiO_2_), presence of fever, C-reactive protein (CRP), renal replacement therapy, BMI, and protein intake as fixed effects. The covariates for the remaining models are summarised in Supplemental Table 4. Continuous data were scaled and centred. Model assumptions and collinearity were checked (Supplemental Fig. 1 and Table 5). Results were presented as predicted energy expenditure with regression coefficients and 95% confidence intervals. Time entered the model as a continuous variable. Non-linear trends over time were modelled using spline functions. The number of knots was selected by Akaike’s Information Criterion (AIC) (Supplemental Table 6). The selected final model was compared to a model without time using analysis of variance.

In an exploratory analysis, LCA was conducted among patients with ≥ 2 measurements. The same LMM was applied as in the primary analyses. Class number selection was based on a combination of the AIC, the Bayesian Information Criterion (BIC), entropy, and clinical interpretability [12]. To improve convergence, site was modelled as a fixed effect, and no random slopes were allowed.

Analyses were performed in R 4.4.0 or later with relevant packages in Supplemental Table 7. Statistical significance was set at *p* ≤ 0.05. No adjustments were made for multiple comparisons, and all secondary analyses should be considered hypothesis-generating rather than confirmatory.

## Results

### Clinical characteristics

**Fig. 1:**
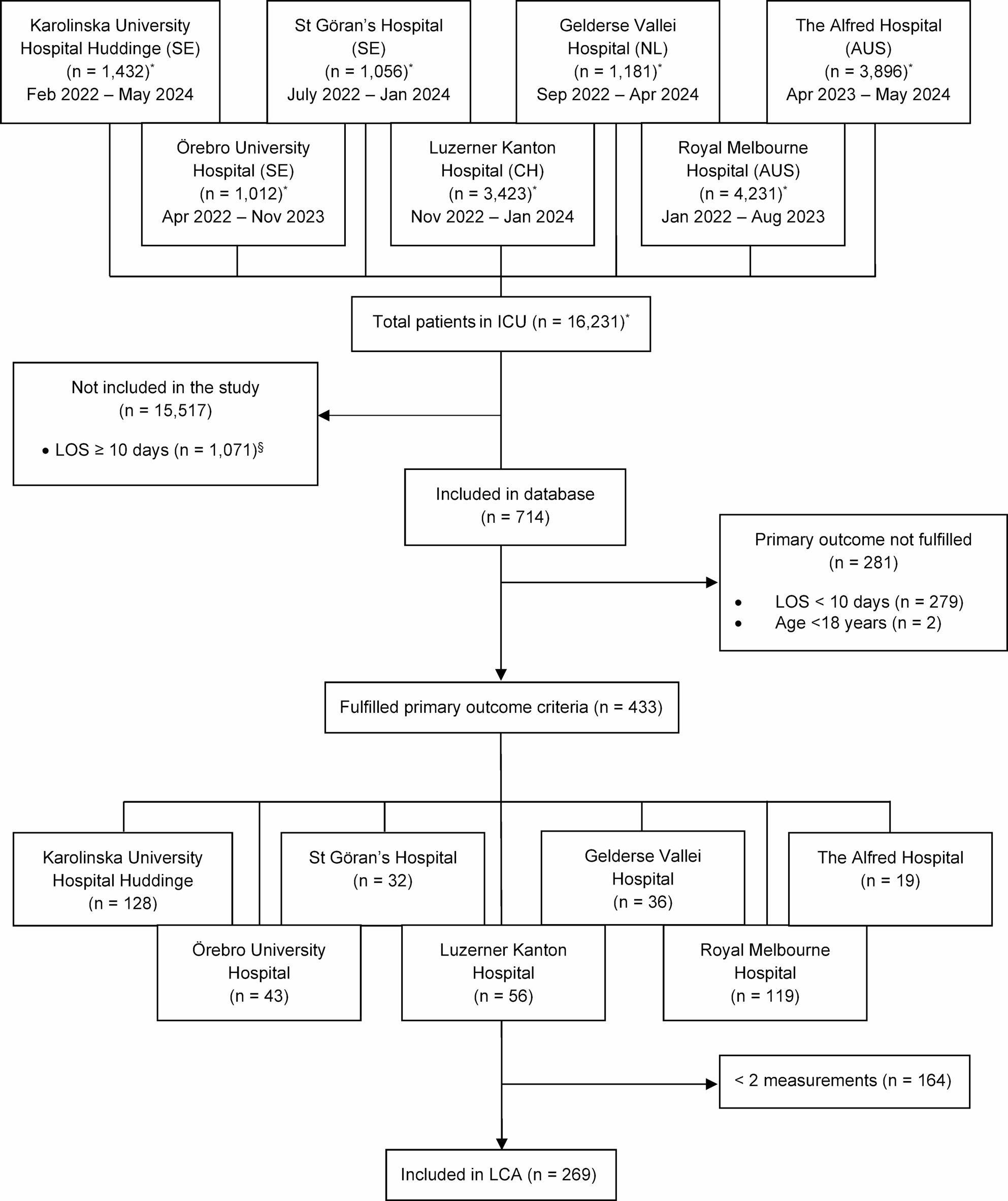
Inclusion flow-chart. For the LCA analysis, only patients with ≥2 measurements were included. *Total number of admissions during the study period at each site. §Reasons for non-inclusion include staff availability, IC availability, and technical reasons. The data for St Göran’s Hospital were extracted from the Swedish Intensive Care Registry post-hoc

Four hundred thirty-three patients were included between January 2022 and May 2024. The screening and inclusion process is summarised in Fig. 1. The mean age was 56 (16) years, and 305 (70%) were male. Of the 35% with surgery before ICU admission, 80% were emergency procedures. The most common diagnostic categories were respiratory (22%), circulatory (15%), and gastrointestinal (14%). Mean SOFA score at admission, available in 227 patients, was 8.1 (3.8). ICU length of stay was 19 days (13–28), and ICU survival was 84%. Baseline characteristics are summarised in Table 1.

**Table 1:** Demographical and clinical description

	Patients *n* = 433
**Patient Characteristics**	
Age (years)	56 (16)
Male (n (%))	305 (70%)
Height (cm)	173 (10)
Weight (kg)	87 (24)
BMI (kg/m^2^)	29 (8)
Admission source (n (%))	
Emergency department	150 (35%)
Ward	117 (27%)
Operating theatre	92 (21%)
Other ICU	74 (17%)
Surgery prior to admission (n (%))^*^	152 (35%)
Emergency surgery (n (%))	121 (80%)
Number of comorbidities (n (SD))	2 (1)
**Illness severity**	
SOFA score at admission (points)	8.1 (3.8), *n* = 227
APACHE II score (points)^†^	19 (8), *n* = 172
APACHE IV score (points)^†^	90 (32), *n* = 16
SAPS II score (points)^‡^	55 (16), *n* = 56
SAPS III score (points)^§^	60 (17), *n* = 200
No prediction score (n (%))	4 (0.9%)
Predicted mortality (%)^||^	27% (23%)
**ICU details**	
Vasopressor during ≥ 1 measurement (n (%))	326 (75%)
Renal replacement therapy during ≥ 1 measurement (n (%))	108 (25%)
Length of stay, incl. previous ICU (days)^**^	19 days (13 days − 28 days)
Sepsis diagnosis during stay (n (%))^††^	
Sepsis	52 (12%)
Septic shock	70 (16%)
No sepsis diagnosis	311 (72%)
Alive at ICU discharge (n (%))	365 (84%)

Statistics presented as mean (SD), number (%) or (SD), or median (Q1 – Q3)

^*^Proportion of the patients with surgery prior to admission

^†^Used by Ziekenhuis Gelderse Vallei, The Royal Melbourne Hospital, and The Alfred Hospital

^‡^Used by Luzerner Kantonsspital

^§^Used by Huddinge University Hospital, St Göran’s Hospital, and Örebro University Hospital

^||^Composite of the different prediction scores used, and calculated using locally validated equations or published equations

^**^Length of stay includes time in previous ICU

^††^According to Sepsis-3 criteria or APACHE II diagnosis codes

BMI = body mass index; ICU = intensive care unit; SOFA = sequential organ failure assessment; APACHE = acute physiology and chronic health evaluation; SAPS = simplified acute physiology score; SD = standard deviation; n = number of non-missing observations

**Fig. 2:**
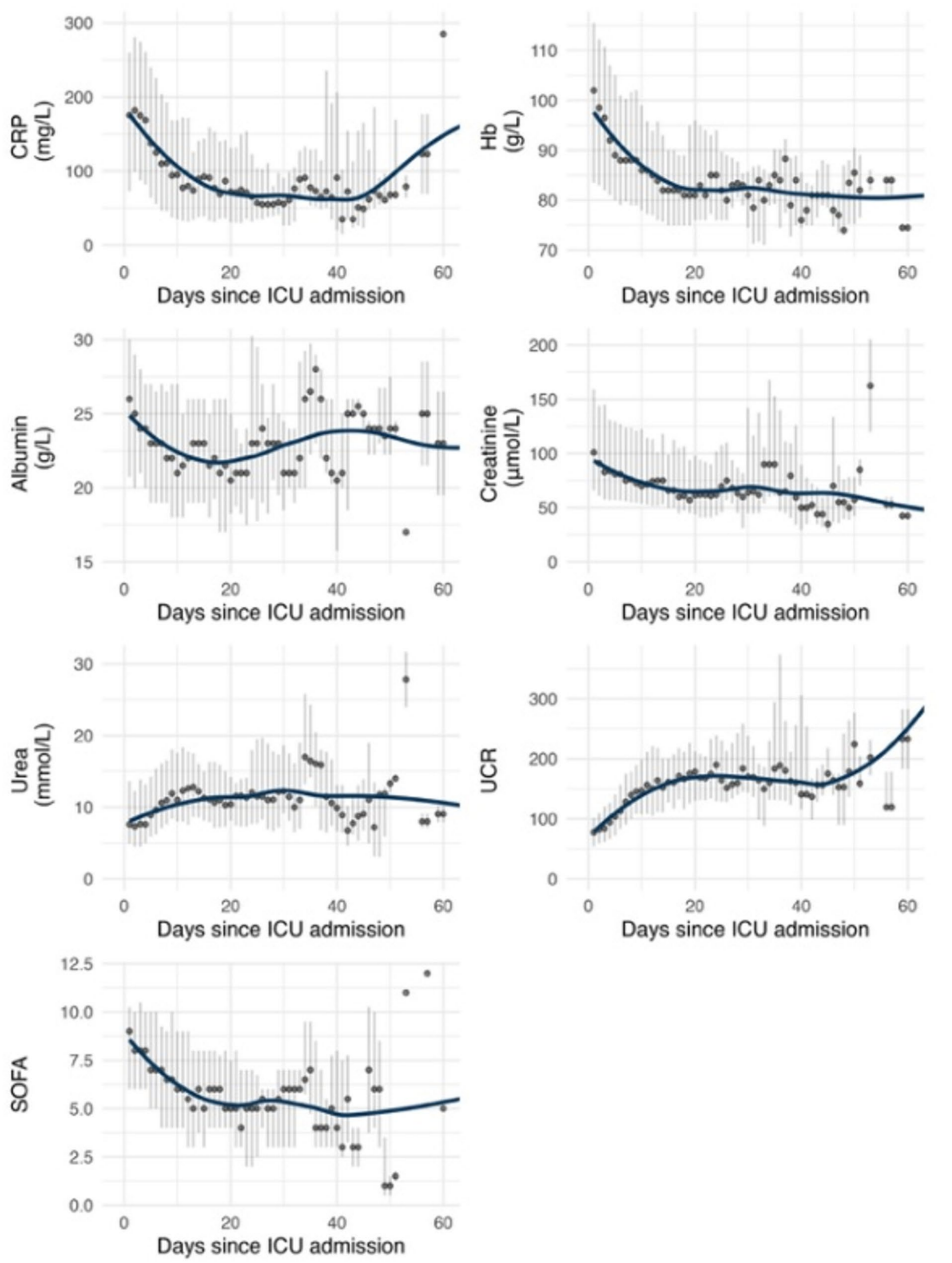
Development of biochemical markers and organ failure. The trajectories of the biochemical markers **(A)** CRP, **(B)** Hemoglobin, **(C)** Albumin, **(D)** Creatinine, **(E)** Urea, and **(F)** Urea: creatinine ratio reflect an expected trajectory during prolonged ICU stay. The UCR rises biphasically. **(G)** SOFA scores at measurement. In A-G, a non-parametric loess smoother with span 0.5 was applied to visualise trends over time. Shaded bars indicate the interquartile range around the 3-day rolling median. CRP = C-reactive protein; Hb = hemoglobin; UCR = urea: creatinine ratio; SOFA = sequential organ failure assessment; ICU = intensive care unit; IQR = interquartile range

A steep increase in the urea: creatinine ratio (UCR) was observed during the first 10 days (*p* < 0.001), which persisted after adjustment for age, CRP, renal replacement therapy, surgery, and carbohydrate intake. Clinical details at each measurement are presented in Table 2, and trajectories of biochemical variables in Fig. 2.

**Table 2:** Indirect calorimetry, nutritional, and clinical data from each measurement

	Measurements *n* = 1194^*^
**Indirect calorimetry**	
Number of measurements per patient (n (%))	2 (2–3)
Day in ICU at measurement number (days):	
1	4 (3–7), *n* = 432
2	9 (6–13), *n* = 341
3	12 (10–18), *n* = 189
4	17 (13–22), *n* = 93
5	21 (15–28), *n* = 55
Day in ICU at measurement (days)	9 (5–16)
Energy expenditure (kcal/day)	1862 (1568–2221)
Energy expenditure / body weight (kcal/kg/day)^†^	25 (21.6–29.1)
Respiratory quotient	0.81 (0.76–0.86)
VO_2_ (ml/min)	271 (226–323)
VCO_2_ (ml/min)	222 (185–260)
**Administered nutrition** ^‡^	
Enteral nutrition (n (%))	1001 (84%)
Kcal / day	1500 (960–1901)
Protein / day (g)	80 (50–109)
Carbohydrates / day (g)	150 (97–194)
Lipids / day (g)	59 (38–76)
Parenteral nutrition (n (%))	196 (16%)
Kcal / day	1173 (899–1682)
Protein / day (g)	65 (48–92)
Carbohydrates / day (g)	121 (92–180)
Lipids / day (g)	44 (34–62)
Glucose infusion (n (%))	415 (35%)
Kcal from glucose infusions / day	53 (29–202)
Amino acid infusion (n (%))	76 (6.4%)
Total protein / body weight (g/kg/day)^†^	1.16 (0.77–1.5)
No nutrition (n (%))	44 (3.7%)
Kcal from propofol / day	288 (185–432)
Lipids from propofol / day (g)	29 (18–43)
Total kcal / day	1729 (1303–2124)
Kcal from carbohydrates (kcal / day)^§^	697 (507–884)
Kcal from protein (kcal / day)^||^	277 (182–385)
Kcal from lipids (kcal / day)^**^	754 (514–1,052)
Total kcal / bodyweight / day (kcal/kg/day)^†^	24 (18–28)
Total kcal / day / energy expenditure (%)	92 (70–110)
**Chemistry at day of measurement**	
Hemoglobin (g/L)	86 (78–100)
CRP (mg/L)	103 (42–187), *n* = 1057
Albumin (g/L)	23 (19–27), *n* = 880
Urea (mmol/L)	10 (7–16), *n* = 1141
Creatinine (µmol/L)	73 (52–120)
Urea: creatinine ratio	134 (89–188), *n* = 1138
**Organ support**	
SOFA score at measurement	6 (4–9), *n* = 681
Mechanical ventilation (n (%))	1193 (100%)
FiO_2_	0.30 (0.25–0.4)
PEEP (cmH_2_O)	8.0 (6.5–10)
Noradrenaline (n (%))	566 (47%)
No vasopressor (n (%))	593 (50%)
Renal replacement therapy (n (%))	233 (20%)
**Analgesia and sedation**	
RASS (n (%))	
Deeply sedated (-5 – -3)	642 (54%)
Mildly sedated (-2–0)	488 (41%)
Agitated (+ 1 – +4)	57 (4.8%)
Propofol (n (%))	612 (51%)
Propofol dose (mg/kg/h)	2.41 (1.53–3.17)
Dexmedetomidine (n (%))	129 (11%)
Thiopentone (n (%))	30 (2.5%)
Ketamine (n (%))	45 (3.8%)
Clonidine (n (%))	112 (9.4%)
No sedative (n (%))	421 (35%)
Epidural analgesia (n (%))	29 (2.4%)
Parenteral opioids (n (%))	797 (67%)
Paracetamol (n (%))	683 (57%)
Temperature > 38.5 °C within 2 h (n (%))	106 (8.9%)

Statistics are presented as number (%) or median (Q1 – Q3)

^*^Number of nonmissing observations is indicated if > 3% of the values were missing

^†^Adjusted body weight if BMI >25

^‡^Calculated values based on rate and energy/macromolecule density at measurement

^§^Includes kcal from glucose

^||^Includes kcal from amino acid infusion

^**^Includes kcal from propofol. SOFA = sequential organ failure assessment; CRP = C-reactive protein; FiO_2_ = fraction of inspired oxygen; PEEP = positive end-expiratory pressure; RASS = Richmond’s agitation and sedation scale; SD = standard deviation

### Changes in energy expenditure and respiratory quotient

In total, 1194 indirect calorimetry measurements were analysed, with 2 (2–3) measurements per patient. Median energy expenditure was 25.0 (21.6–29.1) kcal/kg/day, and RQ was 0.81 (0.76–0.86). Enteral nutrition (EN) was predominant, and total delivery was 24 (18–28) kcal/kg/day, corresponding to 92% (70–100%) of energy expenditure.

**Fig. 3:**
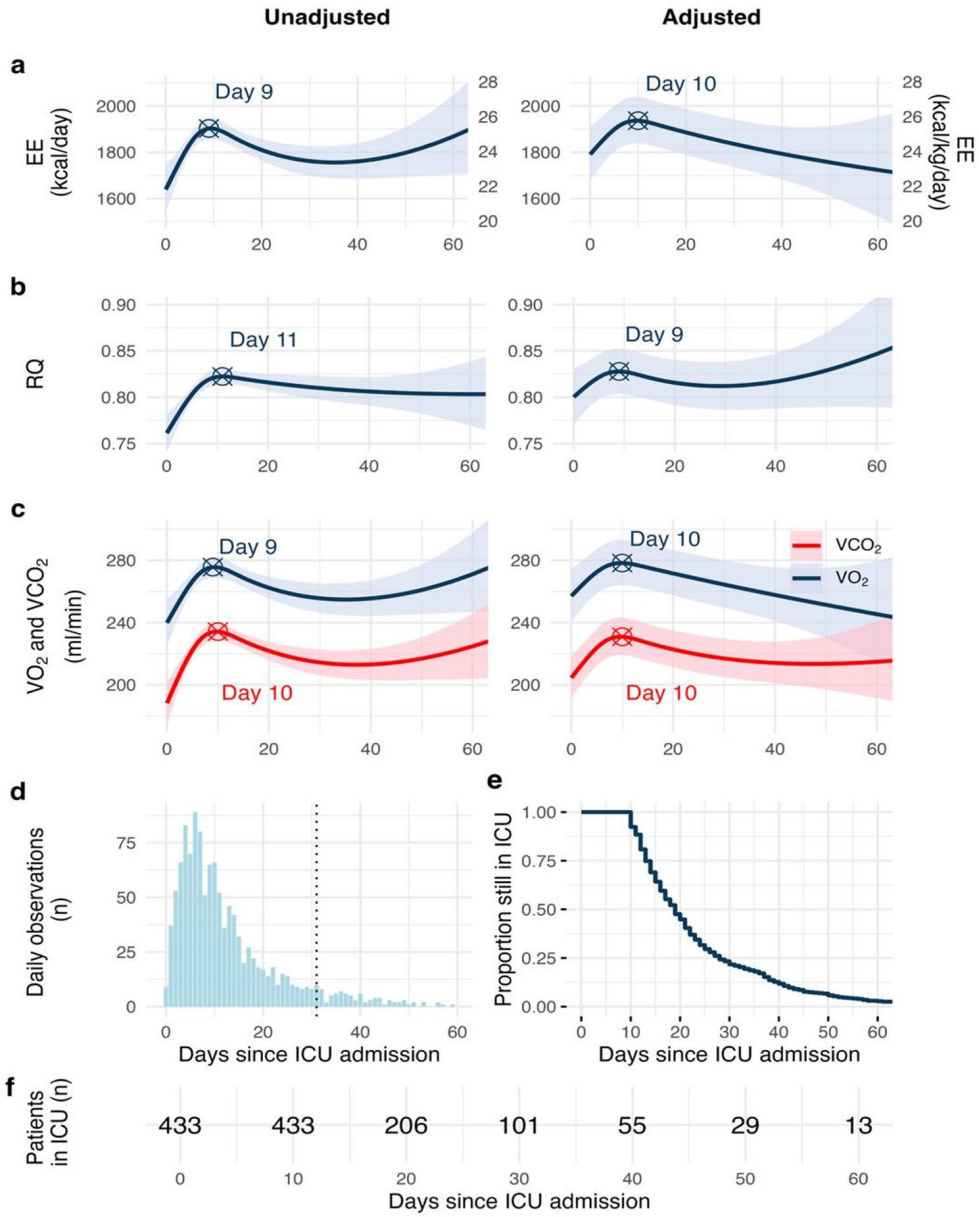
Time course of **A**) energy expenditure, **B**) respiratory quotient, and **C**) VO_2_ and VCO_2_. Predicted energy expenditure, RQ, VO_2_, and VCO_2_ in the unadjusted and adjusted models, respectively. Energy expenditure increased during the first 10 days, and then declined (*p* < 0.01). For the adjusted models, site and patient ID were random effects. In the models for energy expenditure and VO_2_, the fixed effects were time, age, sex, presence of fever, CRP, BMI, and protein and lipid delivery. RQ was adjusted for time, CRP, PEEP, FiO_2_, CRRT, and carbohydrate delivery. VCO_2_ was adjusted for time, age, sex, presence of fever, CRP, BMI, FiO_2_, CRRT, and carbohydrate and energy delivery. **d)** Number of measurements at each day. Beyond day 31 (dotted line), data were sparse (< 10 daily measurements), and this should be considered extrapolation. **e)** Proportion of patients remaining in the ICU at each day. **f)** Number of patients still in the ICU at each day. EE = energy expenditure; RQ = respiratory quotient; VO_2_ = oxygen consumption; VCO_2_ = carbon dioxide production; ICU = intensive care unit; CRP = C-reactive protein; BMI = body mass index; PEEP = positive end-expiratory pressure; FiO_2_ = fraction of inspired oxygen; CRRT = continuous renal replacement therapy

**Fig. 4:**
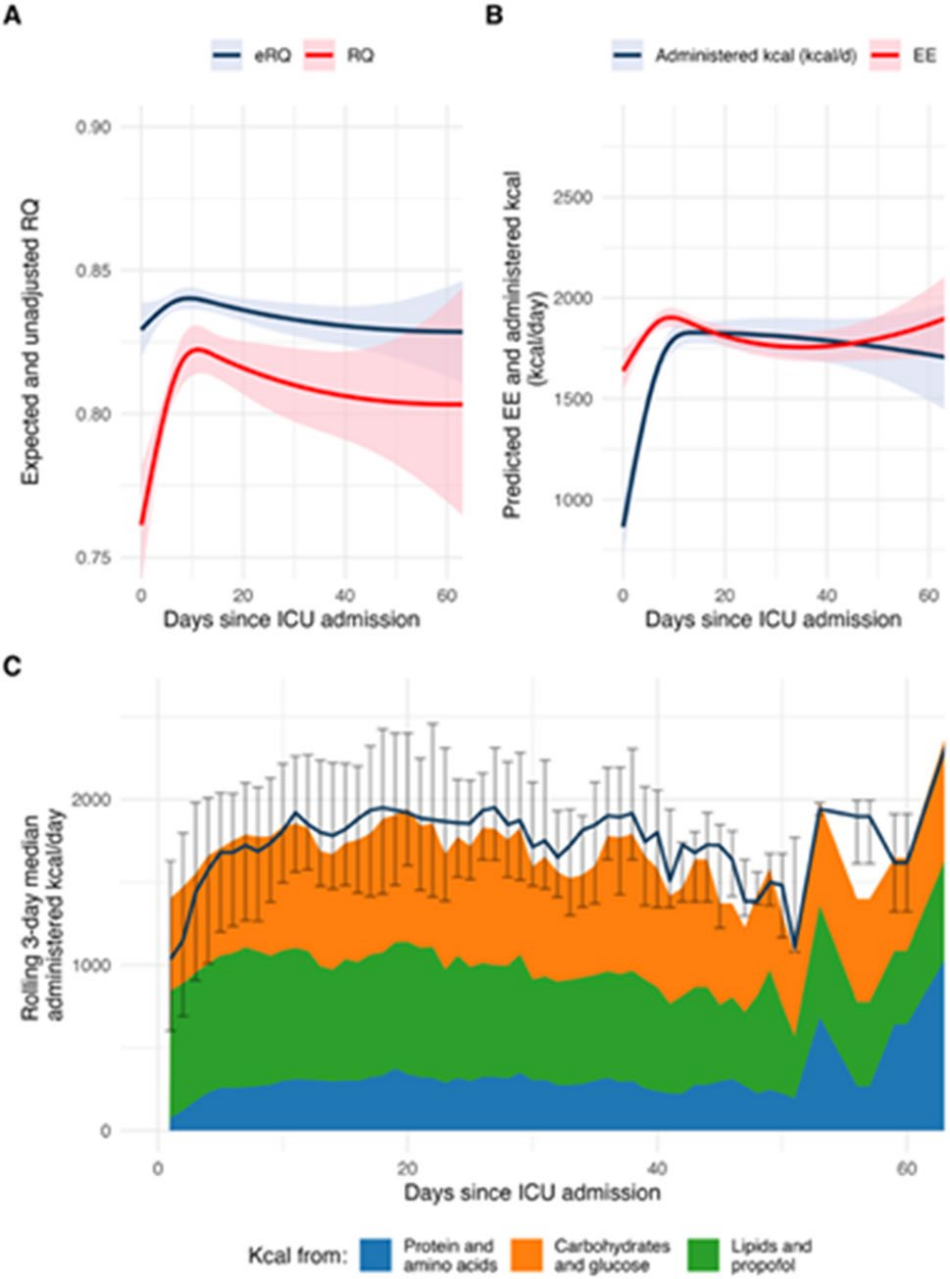
**A**) Measured and expected respiratory quotient. **B**) Measured energy expenditure and administered kcal **C**) Daily macronutrient composition. **A**) The expected vs. the measured RQ. The expected RQ was calculated based on the macronutrient composition in the administered nutrition. During the first 10 days, the measured RQ approaches the expected RQ, suggesting an initial reliance on endogenous substrates. **B**) Measured unadjusted EE vs. the administered kcal/day. **C**) Daily macronutrient composition with total administered kcal. eRQ = expected respiratory quotient; ICU = intensive care unit; EE = energy expenditure; IQR = interquartile range; RQ = respiratory quotient

Energy expenditure exhibited a non-linear trajectory, peaking around day 10 and declining thereafter (Fig. 3A). This remained significant after adjusting for fixed and random effects (*p* = 0.001, conditional R^2^ 0.76). RQ was significantly associated with time, but this lost significance after the inclusion of covariates (*p* = 0.067, conditional R^2^ = 0.53; Fig. 3B). During the first 10 days, RQ approached the expected value for macronutrient intake (Fig. 4A). The amount of administered kcal increased steeply during the first 5 days of the ICU stay, after which it remained stable (Fig. 4B).

### Latent class analysis

**Fig. 5:**
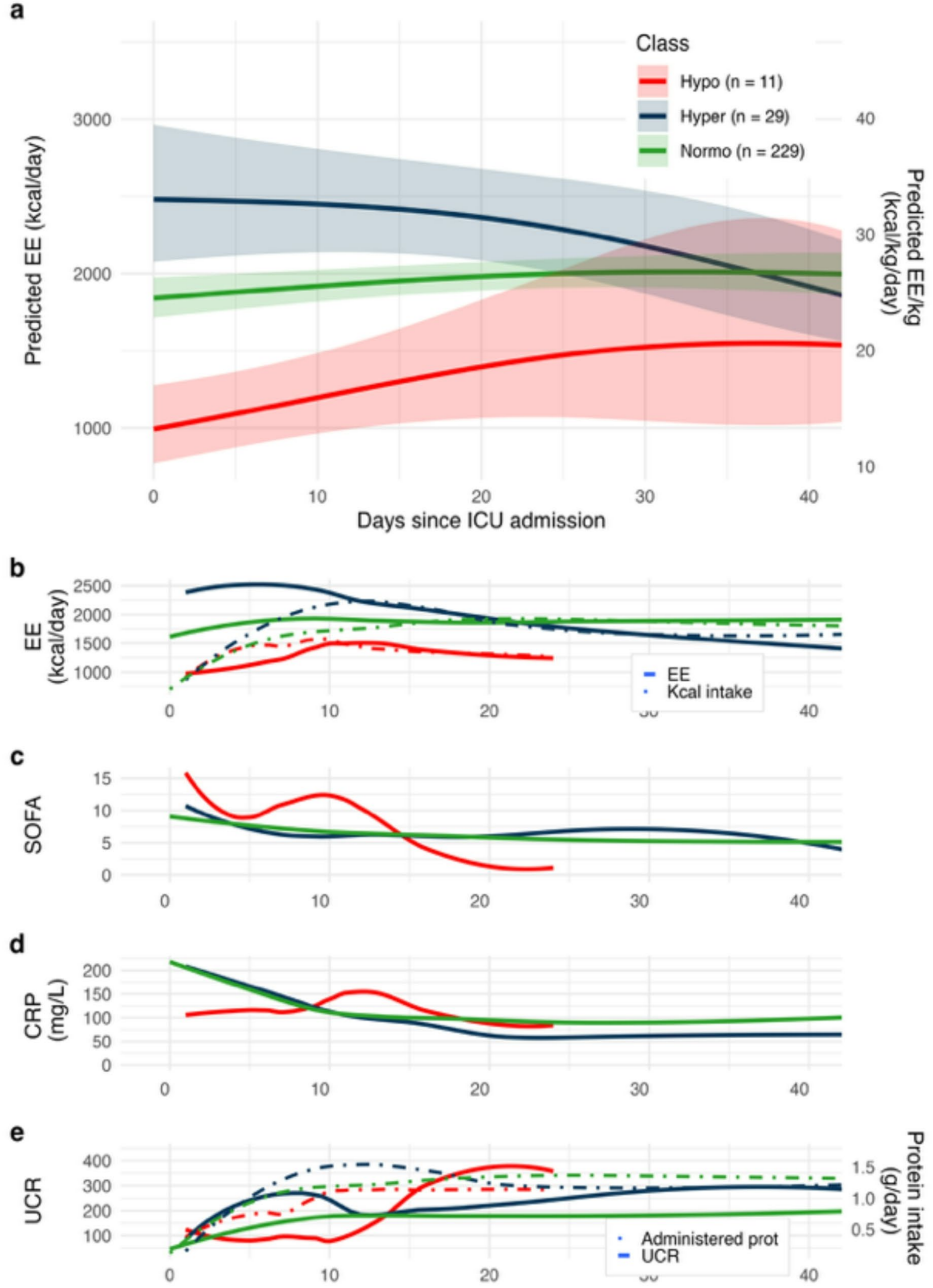
Latent class analysis of energy expenditure trajectories. **(A)** Three trajectories of energy expenditure were found: a hypometabolic class, a normometabolic class, and a hypermetabolic class. **(B)** Class-specific raw trajectories of measured EE (solid lines) and administered kcal (dot-dash line). **C-E)** Class-specific trajectories of SOFA score, CRP, and urea: creatinine ratio, respectively. **B)** The dotted lines indicate the admini4stered kcal/day. **E)** The dotted lines indicate administered protein per day (g/day). Temporal trends visualized using a non-parametric loess smoother. In the hypometabolic class, the last observation was on day 24. EE = energy expenditure; ICU = intensive care unit; SOFA = sequential organ failure assessment score; CRP = C-reactive protein; UCR = urea: creatinine ratio

The LCA included 269 patients with ≥ 2 IC measurements. Three classes with distinct energy expenditure trajectories were identified (Fig. 5A). Entropy in the final model was 0.63. Based on the predicted energy expenditure at ICU day 0, we labelled classes as normo- (*n* = 229; 85%), hyper- (*n* = 29; 11%), and hypometabolic (*n* = 11; 4%). Energy expenditure decreased in the hypermetabolic class but remained stable in the normo- and hypometabolic classes. Due to small class sizes, no inferential analyses were performed and listed class characteristics are purely descriptive.

The hypometabolic class had more males (91%) compared to the hypermetabolic and normometabolic classes (55% and 68%). Mortality prediction and admission SOFA scores were highest in the normometabolic class. In contrast, SOFA scores remained elevated in the hypometabolic class during the first two weeks (Fig. 5B), whereas they decreased in the other groups. ICU mortality was also highest in this class (27% versus 17% and 15%). Temporal patterns of CRP and UCR differed between the groups (Fig. 5C-D).

### Supplementary results, sensitivity and subgroup analyses, missing data

The distribution of ICU diagnoses are presented in Supplemental Fig. 2, and patterns of missing values in Supplemental Fig. 3. Regression coefficients from LMMs for all outcome measures are listed in Supplemental Table 8.

No differences in the time pattern of either energy expenditure, RQ, VO_2_, or VCO_2_ were seen when adding the SOFA score to the models, using only the European patients (Supplemental Fig. 4). The European patients were older, had lower BMI, lower predicted mortality, and more frequently diagnosed with sepsis than the Australian patients (Supplemental Table 9). Data on patients with a length of stay < 10 days (179 patients with 208 measurements) were available from Huddinge University Hospital. To examine whether the inclusion threshold of length of stay ≥ 10 days influenced the trajectory in the acute phase, the short- and long-stay patients were compared, but no significant differences were seen (Supplemental Fig. 5A). Further, we modelled the trajectories using different length of stay thresholds, i.e., the median length of stay of 19 days and the third quantile of 28 days. The biphasic pattern was less prominent in the long-stay models; however, the confidence intervals were overlapping, and no significant differences were seen (Supplemental Fig. 5B). No demographic differences were seen between the patients with the longest length of stay compared to the full cohort (Supplemental Table 10). The trajectory was identical to the primary analysis when analysing only patients with ≥ 2 measurements (Supplemental Fig. 5C). The LCA model selection process and class characteristics are presented in Supplemental Tables 11, 12, and 13.

The frequency of missing data of variables with missingness > 3% is indicated in Tables 1 and 2. CRP was linearly interpolated, which reduced the missingness to 11.5%. The missingness of CRP was assumed to be at random, due to different local routines in sampling. In the final model, 1003 measurements were included in the primary analysis of energy expenditure.

## Discussion

This prospective international observational study investigated trajectories of energy metabolism in patients with persistent critical illness [4, 8]. Our main finding was an early increase in mean energy expenditure during the first 10 days, followed by a gradual attenuation. This pattern was robust after adjusting for covariates. We also observed an early increase in the respiratory quotient, but this trend lost significance after adjustment. Exploratory LCA identified three trajectories of energy expenditure, suggesting distinct metabolic phenotypes within this population.

This is the most extensive prospective study to date describing changes in metabolism in prolonged critical illness. The early trajectory partly aligns with recent observational studies of surgical and mixed ICU cohorts, which have reported an initial increase in energy expenditure during the first week [13–17]. This may reflect treatment-related factors such as increases in feeding and reduced sedation [3]. The pattern also conforms to the conceptual model of a “late-acute” or “flow” phase where energy expenditure is thought to increase after initial resuscitation [2]. The peak observed in our cohort remained statistically significant after adjusting for covariates, indicating a metabolic transition inherent to the trajectory of the acute illness itself. This contrasts with smaller studies in select subgroups of critically ill patients, such as COVID-19 ARDS or severe burns, where persistent hypermetabolism has been reported [18–24]. The variability in reported trajectories highlights the need for systematic synthesis on longitudinal changes in energy expenditure patterns across ICU populations.

The decline in mean energy expenditure in persistent critical illness is a novel finding. Although similar trends have appeared in the aforementioned studies, the number of observations beyond day 10 has been limited. In comparison, this study only included patients remaining in the ICU after day 10, with a median length of stay of 19 days. The associated characteristics of our cohort provide some potential explanations. In line with population-based studies, the strongest predictors of energy expenditure were fundamental biological properties such as age, sex and body mass [25]. Energy expenditure did not appear to be influenced by the degree of organ failure or severity of illness on admission.

Progressive muscle wasting is well described in ICU patients [26]. The urea: creatinine ratio has been proposed as a biomarker of protein wasting in critical illness, reflecting protein catabolism in relation to declining muscle mass [27]. The dynamics of UCR follow those previously observed in persistent critical illness, reaching a plateau of a similar magnitude around day 10 [7, 28]. The concurrent UCR trajectory suggests that protein catabolism and reduced metabolically active mass may have contributed to the decline in energy expenditure over time. As we did not collect longitudinal data on body composition, we are unable to discriminate between a true decline in tissue-specific metabolic rate and a reduction in fat-free mass. While protein overfeeding also elevates UCR, the mean intake in our cohort was moderate despite a subset of Australian patients that were enrolled in the TARGET Protein trial [29].

Another potential explanation of the biphasic trajectory in energy expenditure is that long-term regulation of energy metabolism may be constrained within individual limits. This phenomenon has previously been described in healthy populations [30]. The early increase in energy-consuming biological processes like nutrient-induced thermogenesis or increased respiratory work during weaning efforts may be offset by reduced activity in other functions contributing to basal energy expenditure [30]. However, in the absence of longitudinal body composition data, the relative contribution of such adaptive regulation cannot be determined. To further investigate the relevance of constrained energy expenditure in persistent critical illness and convalescence, studies using doubly labelled water and repeated assessments of fat-free mass would be useful.

Regardless of the causal mechanisms underlying the observed trajectory in energy expenditure, our findings contradict the perception of persistent critical illness as a hypermetabolic state [31]. The observed between-subject variability and variable trajectories reinforces the importance of indirect calorimetry in patients with prolonged ICU stay to avoid over- and underfeeding. Further studies are needed to clarify energy requirements in the post-ICU phase, both before and after hospital discharge.

The observed increase in RQ did not remain significant in the adjusted analysis, including carbohydrate intake as a covariate. Over time, RQ approached the predicted value from exogenous sources. This suggests an early reliance on endogenous lipids and net gluconeogenesis for energy requirements, followed by an increasing utilisation of mixed substrates and exogenous energy sources. As nutrition was gradually introduced over the first week, it was not possible to delineate when the transition from catabolism to a normal physiologic response to feeding occurs. Although the stable plateau in RQ beyond day 10 indicates mixed substrate utilisation from exogenous macronutrients on a group level, there is likely significant heterogeneity in the response to nutrition. Research on biomarkers indicating “readiness” for full feeding would allow for personalised strategies supporting metabolic needs, whereas current recommendations mainly focus on avoiding harm from early overfeeding [32].

Interpretations of the LCA are substantially limited by the small number of observations in the hypo- and hypermetabolic groups. However, our findings indicate directions for future research on the prognostic value of energy expenditure in critical illness. The hypometabolic patients had the highest mortality, the highest SOFA scores over time and a late increase in UCR and CRP. Hypothetically, this may represent an inability to mount a protective physiological response to the primary “hit” in critical illness, including maintaining energy-consuming adaptive metabolic processes, eliciting an inflammatory and immune response, and increasing amino acid turnover through protein breakdown. The lag in elevation of said biomarkers could suggest a secondary insult. Although inference on causal mechanisms was not possible, other studies have found an association between an early depression of energy expenditure and adverse outcomes in sepsis and post-cardiac surgery [33, 34]. This phenomenon could be explained by senescence or a reduction in cardiopulmonary reserve. However, the hypometabolic patients in our study were not older, had similar comorbidities and had the lowest predicted mortality.

To understand the prognostic implications of energy expenditure in this population, a first requisite step is to derive robust cohorts from larger data sets. This would require ubiquitous monitoring of gas exchange in clinical practice, preferably as an integrated part of the ventilator circuit. When regular monitoring is available, combining longitudinal patterns of metabolism with high-throughput metabolomics and transcriptomics could yield valuable insights on metabolic regulation and its interplay with host- and disease-specific factors, both in early and prolonged critical illness [35].

Key strengths of this study are its large sample size, multicentre design across five countries in centres with substantial experience of indirect calorimetry, and high-resolution longitudinal metabolic and nutritional data. Robust statistical modelling, using both prespecified and data-driven approaches, enabled description of population-level trends while accounting for individual heterogeneity. Moreover, the use of LMMs allowed us to analyse trends over time in an unevenly spaced dataset, as well as accounting for systematic differences between sites. The associated characteristics in our cohort align with prior descriptions of persistent critical illness, supporting the applicability to the target population.

This study has several limitations. By design, generalizability is limited to patients remaining in the ICU after 10 days. Patients with shorter stays are likely to be either sicker (and have died) or less ill and discharged earlier. Our cohort comprised 2.7% of cumulative admissions, a smaller fraction than described in population-based studies and registries [11]. This is expected given missed cases and patients for whom indirect calorimetry was not feasible. The actual proportion of admissions with a length of stay *≥* 10 days was 8%, close to our prior assumption.

Second, some variables were calculated to facilitate data collection. Nutritional intake was estimated from formulations and rates provided at measurements, reflecting ongoing rather than daily delivery. This approach was deemed sufficient to capture the diet-induced thermogenic effect [36]. Also, longitudinal data on body composition were not collected.

Third, survivorship bias cannot be excluded. Screening of all eligible patients was not mandated, and reasons for not performing measurements were not documented as they followed clinical practice variations.

These likely include both random (staff availability, workload) and non-random (perceived futility, contraindications) causes. Repeated measurements may therefore have been more frequent in patients expected to survive, partly explaining the discrepancy between predicted and observed mortality. However, this comparison is limited by the use of ICU – rather than hospital - mortality as the observed outcome. Nevertheless, all patients required prolonged invasive ventilation, with SOFA scores and renal replacement rates consistent with severe illness.

Fourth, analyses were not adjusted for informative drop-out, and the decline in energy expenditure after day 10 might be partially driven by attrition. However, no systematic demographic differences were found between patients in the upper quartile of ICU length of stay and others. Observed clinical differences – less vasopressor use, lower haemoglobin and albumin, higher energy delivery – fit expected trajectories of prolonged ICU stay. Similar ICU mortality across groups supports the robustness of the chosen methods.

Fifth, we used an empirical definition of the timing of the onset of persistent critical illness as an inclusion criterion, based on studies on similar clinical populations [4, 8, 37]. The variation of the onset of persistent critical illness might have misclassified some patients, but given that the median length of stay in our cohort was 19 days, we believe that the majority of the included patients had developed persistent critical illness.

Finally, LCA results were strictly exploratory in nature. Entropy was lower than commonly cited thresholds, and the sample size fell short of the recommended > 300 to identify clusters reliably [12]. Limited separation may reflect both small sample sizes and a lack of high-quality indicators. Accordingly, class validity is highly uncertain.

## Conclusions

In this cohort of patients with prolonged ICU stay, energy expenditure followed a biphasic course. An early increase was followed by a gradual decline, coinciding with the onset of persistent critical illness around day 10 in the ICU. Metabolic rate was not substantially elevated, and gas exchange indicated mixed substrate oxidation. Exploratory latent class analysis suggested possible heterogeneity in energy expenditure trajectories, requiring validation in larger cohorts.

## Supplementary Material

Supplementary Material 1.

## Data Availability

The datasets generated and/or analysed during the current study are not publicly available due to the sensitive nature of individuals’ health data, but are available from the corresponding author on reasonable request.

## Abbreviations

AIC: Akaike’s information criterion
BIC: Bayesian information criterion
BMI: Body mass index
CRP: C-reactive protein
EE: Energy expenditure
EN: Enteral nutrition
FiO_2_: Fraction of inspired oxygen
ICU: Intensive care unit
IQR: Interquartile range
LCA: Latent class analysis
LMM: Linear mixed-effects model
LOESS: Locally estimated scatterplot smoothing
PEEP: Positive end-expiratory pressure
RQ: Respiratory quotient
SD: Standard deviation
SOFA: Sequential organ failure assessment score
UCR: Urea: creatinine ratio
VCO_2_: Carbon dioxide production
VO_2_: Oxygen consumption
