# Supplementary material for: Time course of energy expenditure in persistent critical illness: a prospective multicentre study
Source: Crit Care. 2026 May 27;30:273. doi: 10.1186/s13054-026-06102-w (PMC13214077; doi:10.1186/s13054-026-06102-w)
Supplement: Supplementary file 1 — Supplementary Material 1. [file 13054_2026_6102_MOESM1_ESM.docx]

**Time course of energy expenditure in persistent critical illness: an international prospective study**

Timo Oosterveld^1,2^, Michelle C Paulus^3, 4^, Benjamin Hess^5^, Henrike Häbel^6^, Åsa Johansson^7^, Nicole Mürner^5^, Annika Reintam Blaser^5, 8^, Kate Fetterplace^11^, Emma J Ridley^9, 10^, Oana A. Tatucu-Babet^9, 10^, Arthur R H van Zanten^3, 4^, Michael Wanecek^13^, Kym Wittholz^11^, Adam Deane^11, 12^, Olav Rooyackers^1,2^, Martin Sundström Rehal^1,2^

**Online Data Supplement**

Supplemental Table 1

Descriptions of the participating study sites, including, if applicable, ethical permit number, and the indirect calorimeter model used.

| **Hospital** | **Country** | **Ethical permit number** | **Description** | **Indirect calorimeter** |
| --- | --- | --- | --- | --- |
| Karolinska University Hospital Huddinge | Sweden | 2021-02750, Swedish Ethics Review Authority | Tertiary referral hospital with an 8-bed mixed medical-surgical ICU without cardiothoracic/  neurosurgical patients or patients on extracorporeal membrane oxygenation. | Q-NRG (Cosmed, Italy) |
| St Görans Hospital | Sweden | 2021-02750, Swedish Ethics Review Authority | Tertiart referral hospital with mixed ICU. | Q-NRG (Cosmed, Italy) |
| Örebro University Hospital | Sweden | Ändringsansökan, Swedish Ethics Review Authority | Tertiart referral hospital with mixed ICU. | Q-NRG (Cosmed, Italy) |
| Ziekenhuis Gelderse Vallei | The Netherlands | 2205-033, The ethics committee of the Gelderse Vallei Hospital | Regional teaching hospital with two adult ICU units, total of 18 ICU beds. | Q-NRG (Cosmed, Italy) |
| Luzern Canton Hospital | Switzerland | 2022-01575. Ethikkommission Nordwest- und Zentralschweiz (EKNZ). | Tertiary academic hospital with a mixed ICU, total of 24 beds. | Q-NRG (Cosmed, Italy) |
| The Royal Melbourne Hospital | Australia | QA2022008**.** Royal Melbourne Hospital Human Research Ethics Committee (HREC). | Tertiary referral hospital with a 42-bed mixed medical-surgical ICU. | E-sCOVX (GE, Helsinki, Finland) |
| The Alfred Hospital | Australia | Alfred Hospital Ethics Committee; project number 54/23 | The Alfred ICU is a quaternary-level teaching unit, with funding to support up to 67 intensive care beds. | Q-NRG (Cosmed, Italy) |

# **Supplemental Table 2**

| **Measurement standards when performing indirect calorimetry.** |
| --- |
| Accepted instruments for study purposes   - E-sCOVX (General Electric, Helsinki, Finland) - Quark RMR (Cosmed, Rome, Italy) - Q-NRG (Cosmed, Rome, Italy) - BEACON Caresystem (Mermaid Care, Nörresundby, Denmark) |
| Prior to measurement   - The instrument must be calibrated according to manufacturer’s recommendations. Investigators are responsible for keeping records of calibration procedures at the trial site. - The recommended warm-up time for the instrument must be observed before performing a measurement. - The requisite conditions for accurate measurements should be observed for each instrument. Check user´s manual for guidance if FiO_2_ >0.70, PEEP >10, respiratory rate ≥35 and peak inspiratory pressure ≥30 cmH_2_O. - Measurements should be performed under resting conditions and not be preceded by potentially strenuous procedures. The steering group recommends a minimum of one hour’s rest after patient hygiene and three hours after physiotherapy or a painful medical procedure. - FiO_2_, pressure support or tidal volume settings should ideally not be changed one hour prior to performing a measurement. - Measurements should not be performed in the presence of significant leaks in the ventilator circuit or anatomical gas leaks. This should be checked by analysis of ventilator flow waveforms prior to measurements. - Continuous renal replacement therapy may affect the accurate measurement of VCO_2_ but is not a contraindication to performing indirect calorimetry [1]. |
| During the measurement   - Connections to the ventilator circuit should follow manufacturer’s recommendations and avoid excess dead space, which may affect the accuracy of measurements. - FiO_2_, pressure support or tidal volume settings should not be changed during measurements. Suctioning or concurrent delivery of nebulized medications should be avoided. In the event that this occurs, the measurements should be discarded and a new measurement performed at a minimum of one hour later. - If active humidification or an inspiratory filter is used, the sampling point for inspired oxygen fraction should be connected distal to this point to ensure a stable concentration of inspired oxygen. - Measurements should be conducted for a minimum of 15-30 minutes and inspected for stability in VO_2_ and VCO_2_. In general, a variability of <10% is recommended [2]. - Measurements may be performed during mechanical ventilation or using canopy, as per the clinical routines of each site. |
| After the measurement   - Measured resting energy expenditure (REE) and respiratory quotient (RQ) should be documented in the patient’s medical records. Documentation of oxygen consumption (VO2) and carbon dioxide production (VCO2) are ideal but not mandatory. |

# **Supplemental Table 3 – data dictionary.**

All variables used in data management. In the far-right column, an indication as to whether the variable is a calculation based on other variables or not. When applicable, the calculation and/or definition is explained.

| **Variable name** | **Definition** | **Calculated** |
| --- | --- | --- |
| 1 record_id | Unique identifier for record. Assigned by RedCap |  |
| 2 record_meas_id | Unique identifier for measurement within a record. |  |
| 3 redcap_repeat_instance | Number of measurement within a record. |  |
| 4 site | Including site |  |
| 5 meas_date | Date of measurement |  |
| 6 ree | Resting Energy Expenditure |  |
| 7 rq | Respiratory quotient |  |
| 8 vo2 | Oxygen consumption |  |
| 9 vco2 | Carbon dioxide production |  |
| 10 inv_vent | Invasive ventilation |  |
| 11 fio2 | Fraction of inspired oxygen. |  |
| 12 peep | Positive end-expiratory pressure |  |
| 13 bin_sofa_meas_day | Presence of SOFA score at measurement |  |
| 14 sofa_meas_day | SOFA score at measurement |  |
| 15 rass | Richmond’s Agitation and Sedation Scale |  |
| 16 renal_repl | Ongoing renal replacement therapy |  |
| 17 indication_dialysis | If (16) yes, which indication. |  |
| 18 core_temp | Temperature ≥38.5°C within 2 h prior to measurement |  |
| 19 hb | Hemoglobin (g/L) |  |
| 20 crp | C-reactive protein (mcmol/L) |  |
| 21 albumin | Albumin (g/L) |  |
| 22 urea | Urea (mmol/L) |  |
| 23 creatinine | Creatinine (mcmol/L) |  |
| 24 crea_mmol | Creatinine converted to mmol/L | x |
| 25 urea_crea | Urea:creatinine ratio (mmol/L) | x |
| 26 enteral_nutrition | Ongoing enteral nutrition |  |
| 27 parenteral_nutrition | Ongoing parenteral nutrition |  |
| 28 intravenous_glucose | Ongoing glucose infusion |  |
| 29 intravenous_aa | Ongoing amino acid infusion |  |
| 30 no_nutrition | No ongoing nutrition |  |
| 31 nutrition___unk | Unknown nutrition |  |
| 32 cal_dens_ent | Calorie density enteral formula |  |
| 33 prot_dens_ent | Protein density enteral formula |  |
| 34 carb_dens_ent | Carbohydrate density enteral formula |  |
| 35 lip_dens_ent | Lipid density enteral formula |  |
| 36 rate_ent_nutr | Rate enteral formula (ml/h) |  |
| 37 cal_dens_parent | Calorie density parenteral formula |  |
| 38 prot_dens_parent | Protein density parenteral formula |  |
| 39 carb_dens_parent | Carbohydrate density parenteral formula |  |
| 40 lip_dens_parent | Lipid density parenteral formula |  |
| 41 rate_parent_nutr | Rate parenteral formula (ml/h) |  |
| 42 gluc_content | Glucose content (mg/ml) |  |
| 43 gluc_rate | Rate glucose infusion (ml/h) |  |
| 44 prot_aa_inf | Amino acid concentration amino acid infusion |  |
| 45 prot_aa_rate | Rate amino acid infusion |  |
| 46 noradrenaline | Ongoing noradrenaline |  |
| 47 vasopressin | Ongoing vasopressin |  |
| 48 dobutamine | Ongoing dobutamine |  |
| 49 levosimendan | Ongoing levosimendan |  |
| 50 milrinone | Ongoing milrinone |  |
| 51 adrenaline | Ongoing adrenaline |  |
| 52 other_vasopressor | Ongoing other vasopressor |  |
| 53 no_vasopressor | No ongoing vasopressor |  |
| 54 type_vasosupport___unk | Unknown vasopressor |  |
| 55 spec_vasoactive | If (52) yes, which vasopressor |  |
| 56 propofol_10mg | Ongoing propofol infusion with 10 mg/ml |  |
| 57 propofol_20mg | Ongoing propofol infusion with 20 mg/ml |  |
| 58 dexmedetomidine | Ongoing dexmedetomidine |  |
| 59 thiopenthone | Ongoing thiopenthone |  |
| 60 ketamine | Ongoing ketamine |  |
| 61 clonidine | Ongoing clonidine |  |
| 62 other_sedative | Ongoing other sedative |  |
| 63 no_sedative | No ongoing sedative |  |
| 64 type_sed_inf___unk | Sedative unknown |  |
| 65 rate_propofol | Rate propofol (ml/h) |  |
| 66 oth_sed | Other sedative |  |
| 67 epidural | Epidural analgesia in situ |  |
| 68 parenteral_opiods | Parenteral opioids |  |
| 69 paracetamol | Paracetamol |  |
| 70 other_analgesia | Other analgesia |  |
| 71 pain_management___unk | Analgesia unknown |  |
| 72 oth_analg | If (70) yes, which analgesia |  |
| 73 gluc_g_h | Grams glucose per hour (glucose rate * glucose content/1000) | x |
| 74 kcal_ent_h | Kcal delivered enterally per hour (kcal density * EN rate) | x |
| 75 prot_ent_h | Protein (g) delivered enterally per hour (prot density * EN rate) | x |
| 76 carb_ent_h | Carbohydrate (g) delivered enterally per hour (carb density * EN rate) | x |
| 77 lip_ent_h | Lipids (g) delivered enterally per hour (lip density * EN rate) | x |
| 78 kcal_parent_h | Kcal delivered parenterally per hour (kcal density * PN rate) | x |
| 79 prot_parent_h | Protein (g) delivered parenterally per hour (prot density * PN rate) | x |
| 80 carb_parent_h | Carbohydrates (g) delivered parenterally per hour (carb density * PN rate) | x |
| 81 lip_parent_h | Lipids (g) delivered parenterally per hour (lip density * PN rate) | x |
| 82 kcal_glucose_h | Kcal from glucose per hour (gluc_g_h * 3.75) | x |
| 83 kcal_propofol_h | Kcal from propofol per hour (rate_propofol * 1) | x |
| 84 lip_propofol_h | Lipids from propofol (g) per hour (rate_propofol * 0.1) | x |
| 85 prot_aa_h | Amino acids (g) per hour (prot_aa_rate * prot_aa_inf) | x |
| 86 kcal_aa_h | Kcal from amino acid infusion per hour (prot_aa_rate * prot_aa_inf *9) | x |
| 87 gluc_g_24h | Glucose (g) per 24 h (gluc_g_h * 24) | x |
| 88 kcal_ent_24h | Kcal per 24 h delivered enterally (kcal_ent_h * 24) | x |
| 89 prot_ent_24h | Protein (g) per 24 h delivered enterally (prot_ent_h * 24) | x |
| 90 carb_ent_24h | Carbohydrates (g) per 24 h delivered enterally (carb_g_h * 24) | x |
| 91 lip_ent_24h | Lipids (g) per 24 h delivered enterally (lip_ent_h * 24) | x |
| 92 kcal_parent_24h | Kcal per 24 h delivered parenterally (kcal_ent_h * 24) | x |
| 93 prot_parent_24h | Protein (g) per 24 h delivered parenterally (prot_ent_h * 24) | x |
| 94 carb_parent_24h | Carbohydrates (g) per 24 h delivered parenterally (carb_g_h * 24) | x |
| 95 lip_parent_24h | Lipids (g) per 24 h delivered parenterally (lip_ent_h * 24) | x |
| 96 kcal_glucose_24h | Kcal from glucose infusions per 24 h (glucose_kcal_h * 24) | x |
| 97 kcal_propofol_24h | Kcal from propofol infusions per 24 h (kcal_propofol_h * 24) | x |
| 98 lip_propofol_24h | Lipids (g) from propofol infusions per 24 h (lip_propofol_h * 24) | x |
| 99 prot_aa_24h | Protein (g) from amino acid infusion per 24 h (prot_aa_h * 24) | x |
| 100 kcal_aa_24h | Kcal from amino acid infusion per 24 h (kcal_aa_h * 24) | x |
| 101 total_kcal_24h | Total kcal during 24 h (kcal_ent_24h + kcal_parent_24h + kcal_glucose_24h + kcal_propofol_24h + kcal_aa_24h) | x |
| 102 total_prot_24h | Total protein during 24 h (prot_ent_24h + prot_parent_24h + prot_aa_24h) | x |
| 103 total_lip_24h | Total lipids during 24 h (lip_ent_24h + lip_parent_24h + lip_propofol_24h) | x |
| 104 total_carb_24h | Total carbohydrates during 24 h (carb_ent_24h + carb_parent_24h + gluc_g_24h) | x |
| 105 total_kcal_ree | Total administered kcal relative to measured REE (%) (total_kcal_24h / ree * 100) | x |
| 106 fq | Food quotient (0.774*total_prot_24h+1.427*total_lip_24h+0.829*total_carb_24h)/(0.966*total_prot_24h+2.019*total_lip_24h+0.829*total_carb_24h) | x |
| 107 diff_rq_fq | Difference between RQ and FQ | x |
| 108 avg_rq_fq | Average RQ and FQ ((FQ + RQ )/ 2) | x |
| 109 rass_group | RASS grouped: (-5 – -3) ~ 1, (-2 – 0) ~ 2, (1 – 4) ~ 3. | x |
| 110 espen_calc_ree | REE calculated using VCO2 (vco2 * 8.19) | x |
| 111 pcent_glucose_kcal | Percentage of kcal from glucose infusion (kcal_glucose_24h / total_kcal_24h) | x |
| 112 pcent_prop_kcal | Percentage of kcal from propofol (kcal_propofol_24h / total_kcal_24h) | x |
| 113 pcent_pn_kcal | Percentage of kcal from PN (kcal_parent_24h / total_kcal_24h) | x |
| 114 pcent_en_kcal | Percentage of kcal from EN (kcal_ent_24h / total_kcal_24h) | x |
| 115 vasopressor_any | Presence of vasopressor during any measurement within record. | x |
| 116 dialysis_any | Presence of renal replacement during any measurement within record. | x |
| 117 pat_id | Unique pat ID |  |
| 118 dob_pat | Date of birth |  |
| 119 age | Age |  |
| 120 date_reg | Date of registration |  |
| 121 adm_date | Date of admission to ICU |  |
| 122 adm_source | Source of admission |  |
| 123 days_oth_icu | Days in other ICU before admission |  |
| 124 days_bef_oth_icu | Days in hospital before other ICU. |  |
| 125 APACHE_II | Score APACHE II |  |
| 126 APACHE_III | Score APACHE III |  |
| 127 APACHE_IV | Score APACHE IV |  |
| 128 SAPS_III | Score SAPS III |  |
| 129 MPM_III | Score MPM III |  |
| 130 No_pred_score | No prediction score used. |  |
| 131 Pred_tool_other | Other prediction score. |  |
| 132 prediction_score___unk | Unknown prediction score |  |
| 133 score_apache_2 | Score APACHE II. Used by: Ziekenhuis Gelderse Vallei, The Royal Melbourne Hospital, The Alfred Hospital, |  |
| 134 score_apache_4 | Score APACHE IV. Used by: Ziekenhuis Gelderse Vallei (from October 2023 and onward, both APACHE II and IV were used). |  |
| 135 score_saps | Score SAPS III. Used by: Huddinge University Hospital, St Göran’s Hospital, Örebro University Hospital. |  |
| 136 other_pred_tool | If yes (131), other prediction score used. |  |
| 137 score_other_tool | If yes (136), score SAPS II (only SAPS II was in this category). Used by: Lucerne Canton Hospital. |  |
| 138 sofa_adm | SOFA at admission |  |
| 139 surgery | Surgery prior to admission |  |
| 140 emergency_elective | If yes (139), emergency or elective surgery |  |
| 141 sex | Sex |  |
| 142 height | Height (cm) |  |
| 143 weight | Weight (kg) |  |
| 144 adj_bw | Adjusted body weight (If bmi > 25, then (height-100)+0.25*(weight-(height – 100)) | x |
| 145 bmi | BMI (weight/((height/100)^2)) | x |
| 146 Hypertension | Hypertension diagnosis |  |
| 147 Ischemic_heart_disease | Ischemic heart disease diagnosis |  |
| 148 Heart_failure | Heart failure diagnosis |  |
| 149 Diabetes_mellitus | Diabetes diagnosis |  |
| 150 COPD | Chronic obstructive pulmonary disease diagnosis |  |
| 151 Chronic_kidney_disease | Chronic kidney failure |  |
| 152 End_stage_renal_disease | End stage renal disease |  |
| 153 Liver_cirrhosis | Liver cirrhosis |  |
| 154 Active_cancer | Active cancer (solid tumor, not in remission) |  |
| 155 Haematological_malignancy | Hematological malignancy. |  |
| 156 Solid_organ_transplant | Solid organ transplant |  |
| 157 None | No comorbidities |  |
| 158 comorbidity___unk | Unknown comorbidities |  |
| 159 n_comorb | Number of comorbidities | x |
| 160 los | Length of stay |  |
| 161 los_incl_prev_icu | Length of stay including other ICU (if transferred) | x |
| 162 predicted_mortality | Predicted mortality using prediction scores: SAPS ~ exp(-32.06302 + log(score_saps + 10.34171) * 7.199704) /  (1 + exp(-32.06302 + log(score_saps + 10.34171) * 7.199704)),  APACHE II ~ exp(-3.517 + 0.146 * score_apache_2) /  (1 + exp(-3.517 + 0.146 * score_apache_2)),  APACHE IV ~ exp(-5.632 + 0.053 * score_apache_4) /  (1 + exp(-5.632 + 0.053 * score_apache_4)),  SAPS II ~ exp(-7.7631 + 0.0737 * score_other_tool) /  (1 + exp(-7.7631 + 0.0737 * score_other_tool)), | x |
| 163 redcap_data_access_group | Same as site |  |
| 164 disch_date | Discharge date |  |
| 165 surv_status | Alive at discharge |  |
| 166 main_diag | Main diagnosis (ICD-10) |  |
| 167 sepsis | Sepsis, septic shock, no sepsis diagnosis |  |
| 168 length_of_stay | Length of stay |  |
| 169 category | Chapter of ICD-10 classification system | x |
| 170 sub_category | Subcategory/diagnosis in ICD-10 | x |
| 171 respiration | Subcategory/diagnosis in ICD-10 | x |
| 172 circulation | Subcategory/diagnosis in ICD-10 | x |
| 173 gi | Subcategory/diagnosis in ICD-10 | x |
| 174 injury_poisoning | Subcategory/diagnosis in ICD-10 | x |
| 175 infection | Subcategory/diagnosis in ICD-10 | x |
| 176 neoplasms | Subcategory/diagnosis in ICD-10 | x |
| 177 abnormal | Subcategory/diagnosis in ICD-10 | x |
| 178 perinatal | Subcategory/diagnosis in ICD-10 | x |
| 179 neurology | Subcategory/diagnosis in ICD-10 | x |
| 180 health_status | Subcategory/diagnosis in ICD-10 | x |
| 181 other | Subcategory/diagnosis in ICD-10 | x |
| 182 psychiatry | Subcategory/diagnosis in ICD-10 | x |
| 183 endocrine and metabolic diseases | Subcategory/diagnosis in ICD-10 | x |
| 184 musculoskeletal | Subcategory/diagnosis in ICD-10 | x |
| 185 blood disease | Subcategory/diagnosis in ICD-10 | x |
| 186 urology | Subcategory/diagnosis in ICD-10 | x |
| 187 congenital malformations | Subcategory/diagnosis in ICD-10 | x |
| 188 los_at_meas | Length of stay at measurement | x |
| 189 los_at_meas_incl_oth_icu | Length of stay at measurement inlcuding previous ICU | x |
| 190 ree_bw | REE per kg bodyweight (ree / weight) | x |
| 191 ree_adj_bw | REE per kg adjusted bodyweight (ree / adj_bw) | x |
| 192 propofol_dose | Propofol dose (if 10 mg/ml: rate_propofol * 10; if 20 mg/ml: rate_propofol * 20) | x |
| 193 prim_out | LOS ≥10 days | x |
| 194 crp_interp | CRP with interpolated values | x |
| 195 los_fp1_ree | Polynomial term for REE | x |

**Supplemental Table 4**

Prespecified covariates and variable selection process from the statistical plan. The same prespecified variables were used for all models. The other variables were included in the “full” model, i.e., before backwards variable selection, if they correlated significantly with the respective outcome. To account for unbalanced data, the average of the outcome and variable for each patient was used for the correlation analyses.

| **Model** | **Pre-specified variables** | **Significantly correlated variables** |
| --- | --- | --- |
| EE | "age", "sex", "peep", "fio2", "no_vasopressor",  "core_temp", "total_kcal_24h", "rass_group",  "crp_interp", "renal_repl", "bmi", "sepsis", "surgery" | "crp_interp", "creatinine", "total_prot_24h", "total_carb_24h", "total_lip_24h", "propofol_dose" |
| RQ |  | "crp_interp", "urea", "total_prot_24h", "total_carb_24h", "total_lip_24h" |
| VO2 |  | "crp_interp", "propofol_dose", "creatinine", "total_prot_24h", "total_carb_24h", "total_lip_24h" |
| VCO2 |  | "crp_interp", "propofol_dose", "creatinine", "total_prot_24h", "total_carb_24h", "total_lip_24h" |

Supplemental Figure 1

Model diagnostics. The assumptions of the linear mixed-models were assessed using 1) residual plots checking for homoscedasticity, 2) histograms of the residuals checking for normal distribution, and 3) Q-Q plots checking for normality of the residuals. Based on these results, it was concluded that the model assumptions were fulfilled.


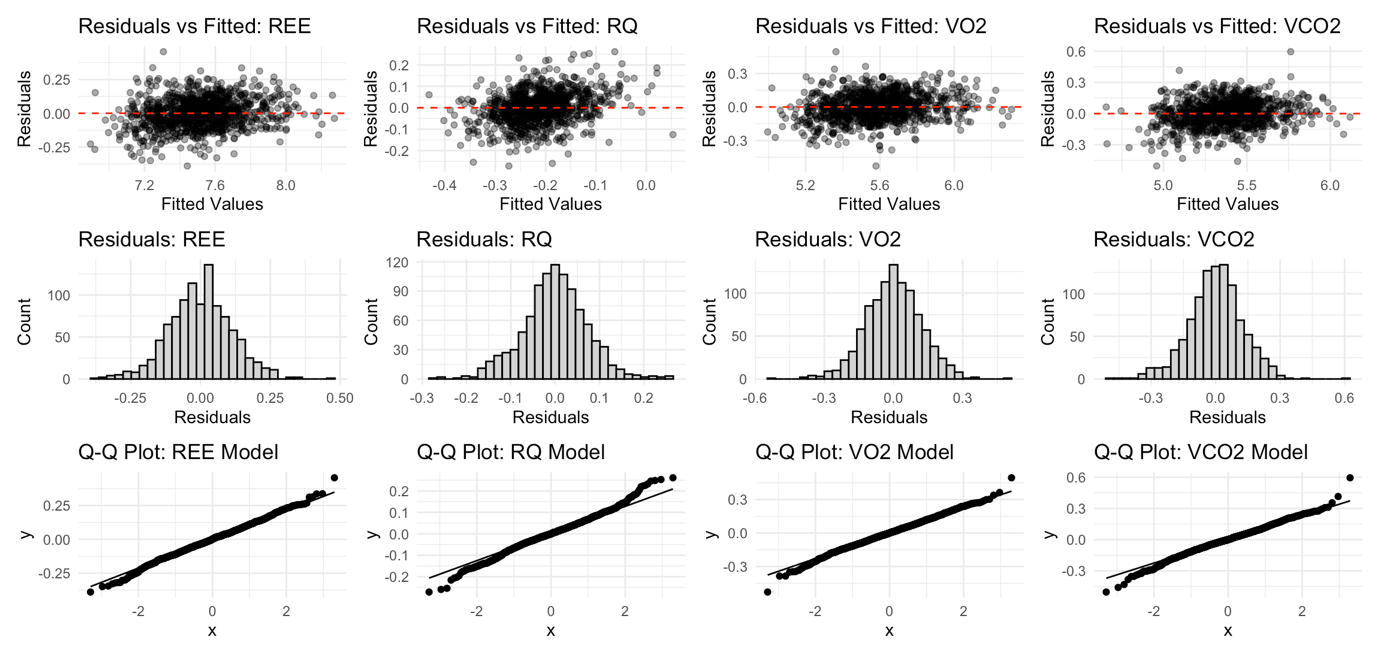


**Supplemental Table 5**

Variance inflation factor for each outcome model. The presence of multicolinearity issues was checked using the variance inflation factor (VIF). Values < 4 were considered acceptable. In the case of a VIF ≥4, the problematic variable was removed from the variable set, and the backwards elimination was repeated.

| **Model** | **Predictor** | **VIF** | **Df** |
| --- | --- | --- | --- |
| REE | ns(los_at_meas_incl_oth_icu, df = 3) | 1.411 | 3 |
| REE | age | 1.043 | 1 |
| REE | sex | 1.048 | 1 |
| REE | Fio2 | 1.069 | 1 |
| REE | core_temp | 1.044 | 1 |
| REE | crp_interp | 1.211 | 1 |
| REE | renal_repl | 1.073 | 1 |
| REE | bmi | 1.034 | 1 |
| REE | total_prot_24h | 1.309 | 1 |
| RQ | ns(los_at_meas_incl_oth_icu, df = 3) | 1.259 | 3 |
| RQ | peep | 1.154 | 1 |
| RQ | fio2 | 1.132 | 1 |
| RQ | crp_interp | 1.188 | 1 |
| RQ | renal_repl | 1.038 | 1 |
| RQ | total_carb_24h | 1.077 | 1 |
| VO2 | ns(los_at_meas_incl_oth_icu, df = 3) | 1.380 | 3 |
| VO2 | age | 1.042 | 1 |
| VO2 | sex | 1.048 | 1 |
| VO2 | core_temp | 1.036 | 1 |
| VO2 | crp_interp | 1.168 | 1 |
| VO2 | bmi | 1.027 | 1 |
| VO2 | total_prot_24h | 1.937 | 1 |
| VO2 | total_lip_24h | 1.701 | 1 |
| VCO2 | ns(los_at_meas_incl_oth_icu, df = 3) | 1.365 | 3 |
| VCO2 | age | 1.041 | 1 |
| VCO2 | sex | 1.053 | 1 |
| VCO2 | fio2 | 1.077 | 1 |
| VCO2 | core_temp | 1.049 | 1 |
| VCO2 | total_kcal_24h | 2.635 | 1 |
| VCO2 | crp_interp | 1.215 | 1 |
| VCO2 | renal_repl | 1.062 | 1 |
| VCO2 | bmi | 1.041 | 1 |
| VCO2 | total_carb_24h | 2.386 | 1 |

Supplemental Table 6

Model selection comparison for the linear mixed models. The Akaike’s information criterion was used to determine the final model. For the model selection, the different approaches to model the time association with each outcome were explored. For all mixed-effects models, the following form was used: log(outcome) ~ time + site + patient-ID. Time was a fixed effect, whereas site and patient were random effects. For each patient, a correlated random slope was allowed. The fractional polynomial model was only explored for the EE.

| **Model Name** | **Description** | **AIC EE** | **AIC RQ** | **AIC VCO_2_** | **AIC V_2_O** |
| --- | --- | --- | --- | --- | --- |
| Linear | Log() ~ linear time + random intercepts | -354 | -2,057 | -229 | -280 |
| Spline, df = 2 | Log() ~ natural spline with 2 df | -373 | -2,061 | -250 | -300 |
| Spline, df = 3 | Log() ~ natural spline with 3 df | **-392** | **-2,066** | **-285** | **-317** |
| Spline, df = 4 | Log() ~ natural spline with 4 df | -384 | -2,062 | -276 | -309 |
| Fractional Polynomial (m_fp) | Log() ~ fractional polynomial of time (selected by mfp) | -389 |  |  |  |
| Piecewise Linear (1 bp at Day 2) | Log() ~ piecewise linear with breakpoint at day 2 | -353 | -2,054 | -232 | -277 |
| Piecewise Linear (1 bp at Day 3) | Log() ~ piecewise linear with breakpoint at day 3 | -362 | -2,054 | -244 | -286 |
| Piecewise Linear (1 bp at Day 4) | Log() ~ piecewise linear with breakpoint at day 4 | -374 | -2,052 | -257 | -299 |
| Piecewise Linear (1 bp at Day 5) | Log() ~ piecewise linear with breakpoint at day 5 | -382 | -2,055 | -268 | -307 |
| Piecewise Linear (1 bp at Day 6) | Log() ~ piecewise linear with breakpoint at day 6 | -385 | -2,056 | -274 | -309 |
| Piecewise Linear (1 bp at Day 7) | Log() ~ piecewise linear with breakpoint at day 7 | -386 | -2,056 | -276 | -310 |
| Piecewise Linear (1 bp at Day 8) | Log() ~ piecewise linear with breakpoint at day 8 | -383 | -2,057 | -274 | -308 |
| Piecewise Linear (1 bp at Day 9) | Log() ~ piecewise linear with breakpoint at day 9 | -379 | -2,057 | -270 | -306 |
| Piecewise Linear (1 bp at Day 10) | Log() ~ piecewise linear with breakpoint at day 10 | -376 | -2,057 | -267 | -303 |
| Piecewise Linear (1 bp) | Log() ~ piecewise linear with 1 breakpoint at 9 | -379 | -2,057 | -270 | -306 |
| Piecewise Linear (2 bp) | Log() ~ piecewise linear with 2 breakpoints at 6, 13 | -373 | -2,045 | -262 | -297 |
| Piecewise Linear (3 bp) | Log() ~ piecewise linear with 3 breakpoints at 5, 9, 16 | -364 | -2,034 | -254 | -289 |
| Piecewise Linear (4 bp) | Log() ~ piecewise linear with 4 breakpoints at 4, 7, 11, 19 | -355 | -2,025 | -246 | -279 |
| Piecewise Linear (5 bp) | Log() ~ piecewise linear with 5 breakpoints at 4, 6, 9, 13, 21.2 | -348 | -2,015 | -238 | -273 |

AIC = Akaike’s information criterion; EE = energy expenditure; RQ = respiratory quotient; VCO_2_ = production of carbon dioxide; VO_2_ = oxygen consumption; df = degree of freedom; bp = breakpoint.

**Supplemental Table 7**

R packages used for the data processing and statistical analyses.

| **Package** | **Version** |
| --- | --- |
| gtsummary | 2.2.0 |
| lme4 | 1.1-37 |
| lmerTest | 3.1-3 |
| gt | 1.0.0 |
| GGally | 2.2.1 |
| tidyverse | 2.0.0 |
| Hmisc | 5.2-3 |
| zoo | 1.8-14 |
| performance | 0.14.0 |
| splines | 4.4.3 |
| ggeffects | 2.2.1 |
| car | 3.1-3 |
| corrr | 0.4.4 |
| flextable | 0.9.7 |
| sjPlot | 2.8.17 |
| lcmm | 2.2.1 |
| patchwork | 1.3.0 |
| mfp | 1.5.4.1 |

**Supplemental Figure 2**

Main ICU diagnoses as per the classification of the International Classification of Diseases, tenth revision (ICD-10).


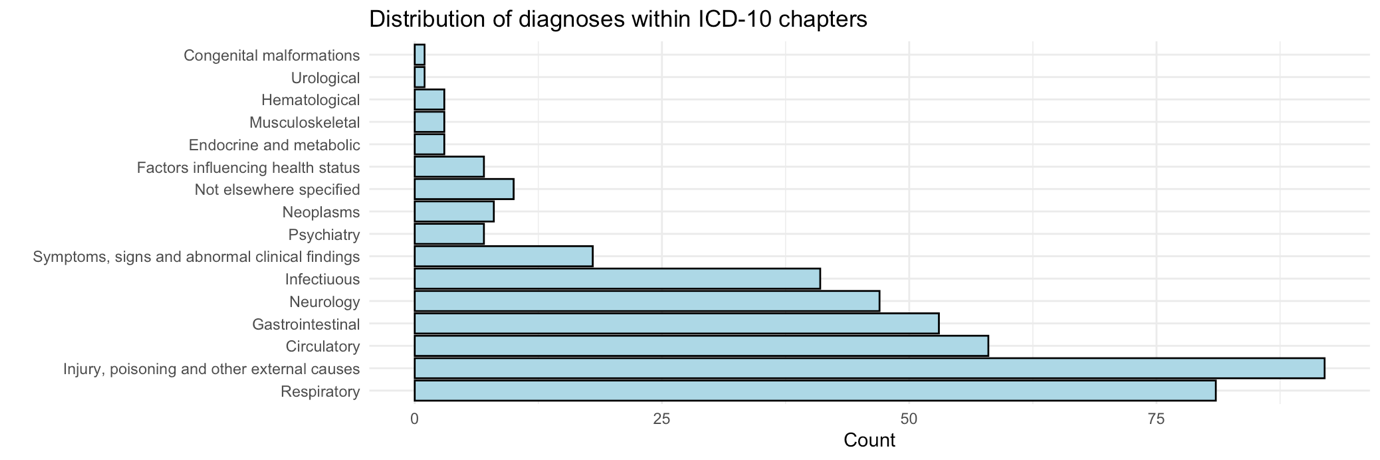


**Supplemental Figure 3**

Missing data pattern of primary outcomes and biochemical variables**.** The light blue bars represent the number of available observations on each day in the ICU. The dark areas represent the number of missing observations. The total number of observations for each day is the sum of the light and dark areas.

**
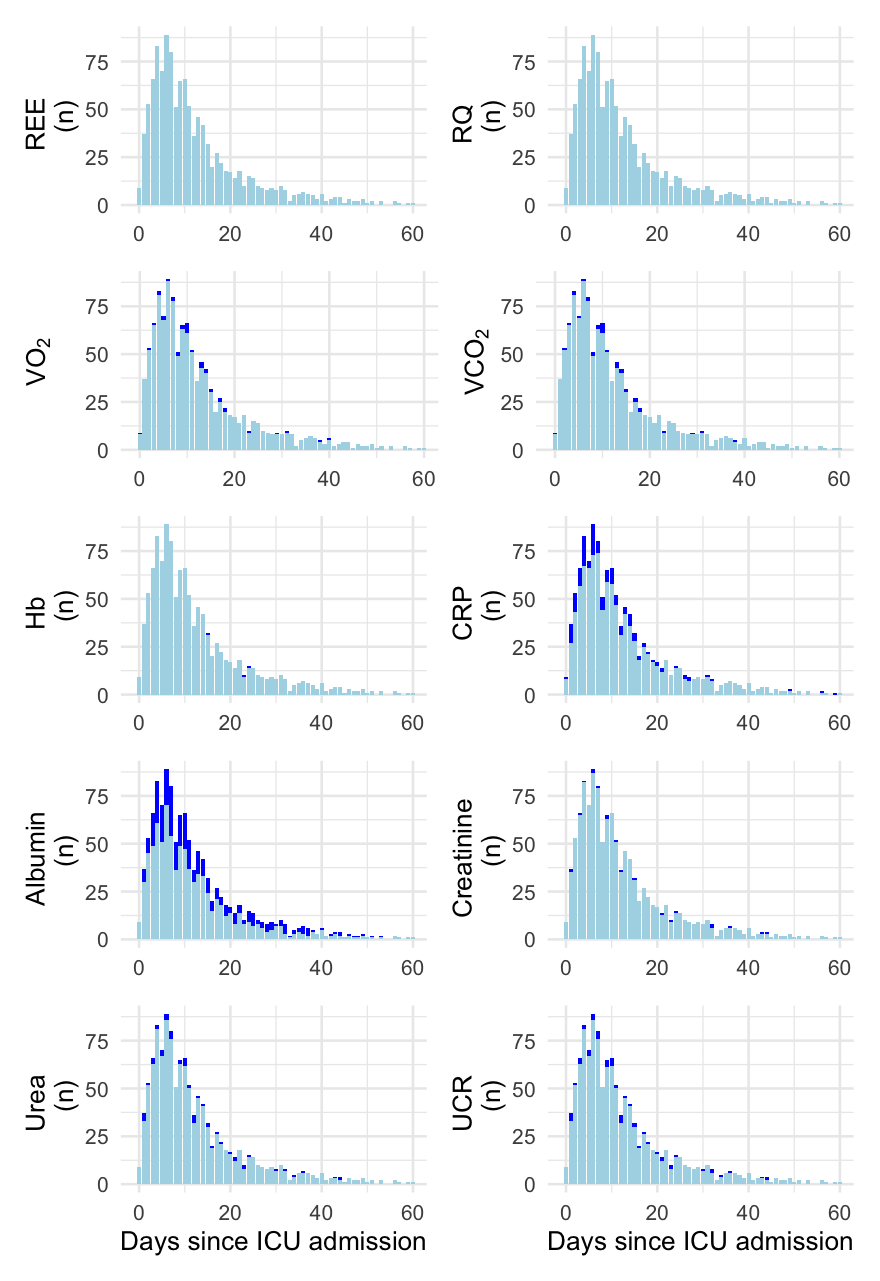
**

**Supplemental Table 8**

Regression table with the results from the linear mixed-effects models. Coefficients have been backtransformed from the log-scale. Variables were centred and scaled prior to analysis. **a** EE and RQ. **b** VO_2_ and VCO_2_.

a

|  | **EE** | | **RQ** | |
| --- | --- | --- | --- | --- |
| *Predictors* | *Estimates* | *p* | *Estimates* | *p* |
| (Intercept) | 1739.05 (1621.81 – 1864.75) | **<0.001** | 0.82 (0.79 – 0.85) | **<0.001** |
| los at meas incl oth icu [1st degree] | 0.96 (0.88 – 1.05) | 0.342 | 0.95 (0.91 – 1.00) | 0.071 |
| los at meas incl oth icu [2nd degree] | 1.03 (0.87 – 1.23) | 0.725 | 1.09 (0.99 – 1.20) | 0.085 |
| los at meas incl oth icu [3rd degree] | 0.88 (0.66 – 1.18) | 0.392 | 1.13 (0.95 – 1.34) | 0.161 |
| age | 0.94 (0.93 – 0.96) | **<0.001** |  |  |
| sex [Female] | 0.85 (0.82 – 0.88) | **<0.001** |  |  |
| fio2 | 1.01 (1.00 – 1.03) | **0.040** | 1.02 (1.01 – 1.03) | **<0.001** |
| core temp [Yes] | 1.10 (1.06 – 1.14) | **<0.001** |  |  |
| crp interp | 1.00 (1.00 – 1.00) | **<0.001** | 1.00 (1.00 – 1.00) | **<0.001** |
| renal repl [Yes] | 0.96 (0.93 – 1.00) | **0.027** | 0.98 (0.96 – 1.00) | **0.035** |
| bmi | 1.11 (1.10 – 1.13) | **<0.001** |  |  |
| total prot 24h | 1.05 (1.04 – 1.06) | **<0.001** |  |  |
| peep |  |  | 0.99 (0.98 – 1.00) | **0.021** |
| total carb 24h |  |  | 1.03 (1.02 – 1.03) | **<0.001** |
| **Random Effects** | | | | |
| σ^2^ | 0.02 | | 0.01 | |
| τ_00_ | 0.03 _record_id_ | | 0.01 _record_id_ | |
|  | 0.00 _site_ | | 0.00 _site_ | |
| τ_11_ | 0.00 _record_id.los_at_meas_incl_oth_icu_ | | 0.00 _record_id.los_at_meas_incl_oth_icu_ | |
| ρ_01_ | -0.82 _record_id_ | | -0.81 _record_id_ | |
| ICC | 0.62 | | 0.48 | |
| N | 393 _record_id_ | | 395 _record_id_ | |
|  | 7 _site_ | | 7 _site_ | |
| Observations | 1003 | | 978 | |
| Marginal R^2^ / Conditional R^2^ | 0.373 / 0.764 | | 0.095 / 0.530 | |

b

|  | **VO_2_** | | **VCO_2_** | |
| --- | --- | --- | --- | --- |
| *Predictors* | *Estimates* | *p* | *Estimates* | *p* |
| (Intercept) | 247.56 (230.48 – 265.91) | **<0.001** | 200.27 (186.56 – 214.99) | **<0.001** |
| los at meas incl oth icu [1st degree] | 0.97 (0.89 – 1.06) | 0.498 | 0.94 (0.86 – 1.02) | 0.154 |
| los at meas incl oth icu [2nd degree] | 1.02 (0.85 – 1.22) | 0.821 | 1.19 (1.01 – 1.40) | **0.041** |
| los at meas incl oth icu [3rd degree] | 0.85 (0.63 – 1.16) | 0.319 | 1.03 (0.79 – 1.36) | 0.806 |
| age | 0.95 (0.93 – 0.96) | **<0.001** | 0.95 (0.94 – 0.97) | **<0.001** |
| sex [Female] | 0.86 (0.83 – 0.89) | **<0.001** | 0.86 (0.83 – 0.89) | **<0.001** |
| core temp [Yes] | 1.10 (1.06 – 1.15) | **<0.001** | 1.10 (1.06 – 1.15) | **<0.001** |
| crp interp | 1.00 (1.00 – 1.00) | **<0.001** | 1.00 (1.00 – 1.00) | **0.013** |
| bmi | 1.11 (1.09 – 1.13) | **<0.001** | 1.10 (1.09 – 1.12) | **<0.001** |
| total prot 24h | 1.03 (1.02 – 1.05) | **<0.001** |  |  |
| total lip 24h | 1.02 (1.00 – 1.03) | **0.019** |  |  |
| fio2 |  |  | 1.03 (1.01 – 1.04) | **<0.001** |
| total kcal 24h |  |  | 1.05 (1.03 – 1.07) | **<0.001** |
| renal repl [Yes] |  |  | 0.96 (0.92 – 0.99) | **0.010** |
| total carb 24h |  |  | 1.02 (1.00 – 1.04) | **0.023** |
| **Random Effects** | | | | |
| σ^2^ | 0.02 | | 0.02 | |
| τ_00_ | 0.03 _record_id_ | | 0.02 _record_id_ | |
|  | 0.00 _site_ | | 0.00 _site_ | |
| τ_11_ | 0.00 _record_id.los_at_meas_incl_oth_icu_ | | 0.00 _record_id.los_at_meas_incl_oth_icu_ | |
| ρ_01_ | -0.78 _record_id_ | | -0.81 _record_id_ | |
| ICC | 0.60 | | 0.57 | |
| N | 392 _record_id_ | | 393 _record_id_ | |
|  | 7 _site_ | | 7 _site_ | |
| Observations | 1001 | | 1003 | |
| Marginal R^2^ / Conditional R^2^ | 0.374 / 0.748 | | 0.370 / 0.727 | |

**Supplemental Figure 4**

Comparison of models with and without SOFA score (SOFA subgroup analysis). In the patients with SOFA score available SOFA scores were added to the model, but were neither associated with EE, RQ, VO_2_, or VCO_2_, nor did they change the time-outcome relationships in primary analyses.

The time association between the outcomes (EE, RQ, VO_2_, and VCO_2_, respectively) remained unchanged. Note, for VO_2_ and VCO_2_, there were no observations after day 37. EE = energy expenditure; ICU = intensive care unit.


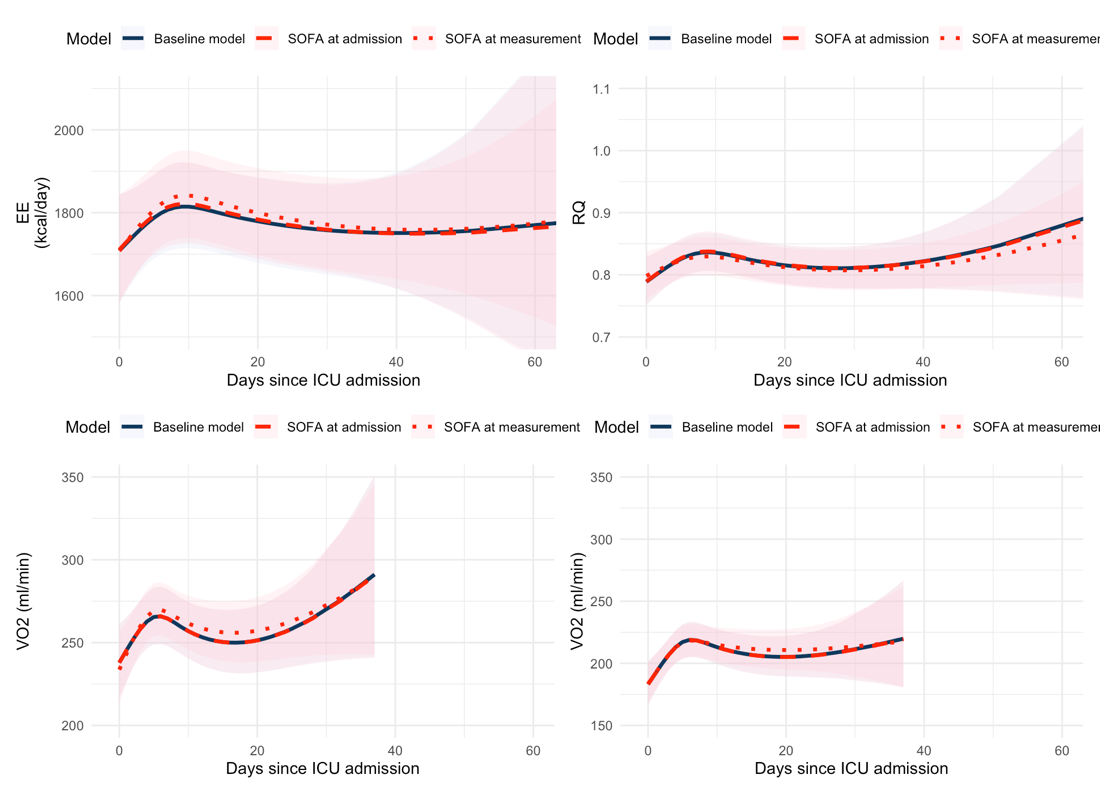


**Supplemental Table 9 – Australian vs non-Australian patients**

Comparison of demographic (9A) and clinical details at each measurement (9B) of the Australian patients and the non-Australian patients.

|  | **AUS vs non-AUS** | | |
| --- | --- | --- | --- |
|  | **Australian**  N = 138*^1^* | **Non-Australian**  N = 295*^1^* | **p-value***^2^* |
| **Patient Characteristics** | | | |
| Age (years) | 49 (16) | 60 (15) | **<0.001** |
| Male | 99 (72%) | 206 (70%) | 0.7 |
| Height (cm) | 173 (11) | 173 (10) | 0.5 |
| Weight (kg) | 92 (28) | 85 (22) | **0.034** |
| BMI (kg/m^2^) | 31 (9) | 28 (7) | **0.011** |
| Admission source |  |  | **<0.001** |
| Emergency department | 77 (56%) | 73 (25%) |  |
| Ward | 23 (17%) | 94 (32%) |  |
| Operating theatre | 35 (25%) | 57 (19%) |  |
| Other ICU | 3 (2.2%) | 71 (24%) |  |
| Surgery prior to admission | 51 (37%) | 101 (34%) | 0.6 |
| Emergency surgery | 44 (86%), n = 51 | 77 (76%), n = 101 | 0.15 |
| Number of comorbidities | 1.72 (1.01) | 2.16 (1.13) | **<0.001** |
| **Severity** | | | |
| SOFA score at admission | NA (NA), n = 0 | 8.1 (3.8), n = 227 |  |
| APACHE II score | 18 (7), n = 136 | 23 (8), n = 36 | **0.002** |
| APACHE IV score | NA (NA), n = 0 | 90 (32), n = 16 |  |
| SAPS III score | NA (NA), n = 0 | 60 (17), n = 200 |  |
| SAPS II score | NA (NA), n = 0 | 55 (16), n = 56 |  |
| Predicted mortality | 0.33 (0.20) | 0.24 (0.24) | **<0.001** |
| No prediction score | 2 (1.4%) | 2 (0.7%) | 0.6 |
| **ICU details** | | | |
| Vasopressor during ≥1 measurement | 101 (73%) | 225 (76%) | 0.5 |
| Dialysis during ≥1 measurement | 35 (25%) | 73 (25%) | 0.9 |
| Length of stay, incl. previous ICU | 20 days (14 days - 28 days) | 18 days (13 days - 29 days) | 0.3 |
| Sepsis diagnosis during stay |  |  | **<0.001** |
| Sepsis | 13 (9.4%) | 39 (13%) |  |
| Septic shock | 2 (1.4%) | 68 (23%) |  |
| No sepsis diagnosis | 123 (89%) | 188 (64%) |  |
| Alive at ICU discharge | 114 (83%) | 251 (85%) | 0.5 |
| *^1^*Mean (SD); n (%); Mean (SD), n = N Non-missing; n (%), n = N; Median (Q1 - Q3) | | | |
| *^2^*Wilcoxon rank sum test; Pearson's Chi-squared test; Fisher's exact test | | | |
|  | | | |

|  | **AUS vs non-AUS** | | |
| --- | --- | --- | --- |
|  | **Australian**  N = 394*^1^* | **Non-Australian** N = 800*^1^* | **p-value***^2^* |
| Indirect calorimetry | | | |
| Day at measurement | 9 (5 – 14) | 10 (5 – 17) | **0.017** |
| Resting energy expenditure (kcal/day) | 2,092 (1,750, 2,592) | 1,805 (1,511, 2,088) | **<0.001** |
| Resting energy expenditure/adjusted body weight (kcal/kg/day) | 27.5 (24.3, 32.4) | 23.7 (20.9, 27.9) | **<0.001** |
| Respiratory quotient | 0.81 (0.77, 0.86) | 0.80 (0.75, 0.87) | 0.14 |
| VO_2_ | 306 (250, 375) | 261 (219, 304) | **<0.001** |
| VCO_2_ | 246 (210, 303) | 211 (178, 247) | **<0.001** |
| Administered nutrition | | | |
| Enteral nutrition | 338 (86%) | 663 (83%) | 0.2 |
| Kcal / day | 1,788 (657) | 1,301 (624) | **<0.001** |
| Prot / day (g) | 109 (51) | 70 (34) | **<0.001** |
| Carbohydrates / day (g) | 172 (82) | 134 (69) | **<0.001** |
| Lipids / day (g) | 74 (31) | 51 (27) | **<0.001** |
| Parenteral nutrition | 42 (11%) | 154 (19%) | **<0.001** |
| Kcal / day | 1,642 (593) | 1,180 (513) | **<0.001** |
| Protein / day (g) | 90 (30) | 64 (31) | **<0.001** |
| Carbohydrates / day (g) | 193 (73) | 131 (101) | **<0.001** |
| Lipids / day (g) | 62 (22) | 44 (19) | **<0.001** |
| Glucose infusion | 44 (11%) | 371 (46%) | **<0.001** |
| Kcal from glucose infusions / day | 81 (188) | 174 (212) | **<0.001** |
| Amino acid infusion | 2 (0.5%) | 74 (9.3%) | **<0.001** |
| Protein g/kg/day | 1.34 (0.67) | 1.03 (0.55) | **<0.001** |
| No nutrition | 27 (6.9%) | 17 (2.1%) | **<0.001** |
| Kcal from propofol / day | 369 (178) | 270 (131) | **<0.001** |
| Lipids from propofol / day (g) | 37 (18) | 27 (13) | **<0.001** |
| Total kcal / day | 2,000 (723) | 1,580 (612) | **<0.001** |
| Total kcal / day / REE (%) | 91 (34) | 88 (34) | **0.030** |
| SOFA, CRRT, fever | | | |
| SOFA score at measurement | NA (NA, NA), n = 0 | 6 (4, 9), n = 681 |  |
| Temperature >38.5°C within 2 h | 32 (8.1%) | 74 (9.3%) | 0.5 |
| Continuous renal replacement therapy | 75 (19%) | 158 (20%) | 0.8 |
| Chemistry | | | |
| Hemoglobin (g/L) | 88 (15) | 91 (17) | **<0.001** |
| CRP (mcmol/L) | 122 (94) | 130 (108) | 0.6 |
| Albumin (g/L) | 23.8 (5.3) | 22.9 (6.0) | **0.008** |
| Urea (mcmol/L) | 12 (8) | 13 (10) | **0.005** |
| Creatinine (mcmol/L) | 109 (95) | 100 (83) | 0.10 |
| Urea:creatinine ratio | 139 (236) | 173 (258) | **<0.001** |
| Ventilation and circulation | | | |
| Invasive ventilation | 394 (100%) | 799 (100%) | >0.9 |
| FiO_2_ | 0.30 (0.08) | 0.34 (0.11) | **<0.001** |
| PEEP (cmH_2_O) | 9.1 (2.3) | 8.1 (2.3) | **<0.001** |
| Noradrenaline | 175 (44%) | 391 (49%) | 0.15 |
| No vasopressor | 208 (53%) | 385 (48%) | 0.13 |
| Analgesia and sedation | | | |
| RASS |  |  | **<0.001** |
| Deeply sedated (-5 - -3) | 234 (60%) | 408 (51%) |  |
| Mildly sedated (-2 - 0) | 123 (32%) | 365 (46%) |  |
| Agitated (+1 - +4) | 33 (8.5%) | 24 (3.0%) |  |
| Propofol dose (mg/kg/h) | 2.00 (0.94), n = 234 (59%) | 1.51 (0.69), n = 378 (47%) | **<0.001** |
| Dexmedetomidine | 40 (10%) | 89 (11%) | 0.6 |
| Thiopenthone | 27 (6.9%) | 3 (0.4%) | **<0.001** |
| Ketamine | 30 (7.6%) | 15 (1.9%) | **<0.001** |
| Clonidine | 2 (0.5%) | 110 (14%) | **<0.001** |
| No sedative | 116 (29%) | 305 (38%) | **0.003** |
| Epidural analgesia | 3 (0.8%) | 26 (3.3%) | **0.009** |
| Parenteral opioids | 232 (59%) | 565 (71%) | **<0.001** |
| Paracetamol | 171 (43%) | 512 (64%) | **<0.001** |
|  | | | |
| los_at_meas_incl_oth_icu | 12 (10) | 13 (12) | **0.017** |
| *^1^*n (%); Mean (SD); Median (Q1, Q3), n = N Non-missing; Mean (SD), n = N Non-missing (% Non-missing); Median (Q1, Q3) | | | |
| *^2^*Fisher's exact test; Wilcoxon rank sum test; Pearson's Chi-squared test | | | |

**Supplemental Figure 5**

Sensitivity analyses. **A.** For patients from Huddinge University Hospital, data was available for patients with LOS ≤10 days. These were compared to patients from Huddinge University Hospital with LOS > 10 days. **B.** Modelling of energy expenditure for patients with LOS 10-27 days, >18 days, and >28 days. **C.** Modelling of energy expenditure for all patients and only patients with repeated measurements. LOS = length of stay; HS = Huddinge Hospital; EE = energy expenditure; ICU = intensive care unit.


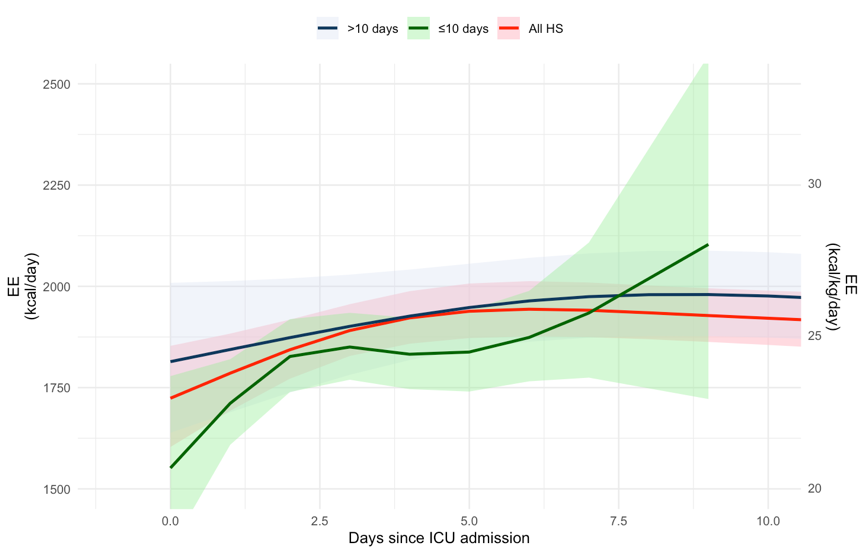

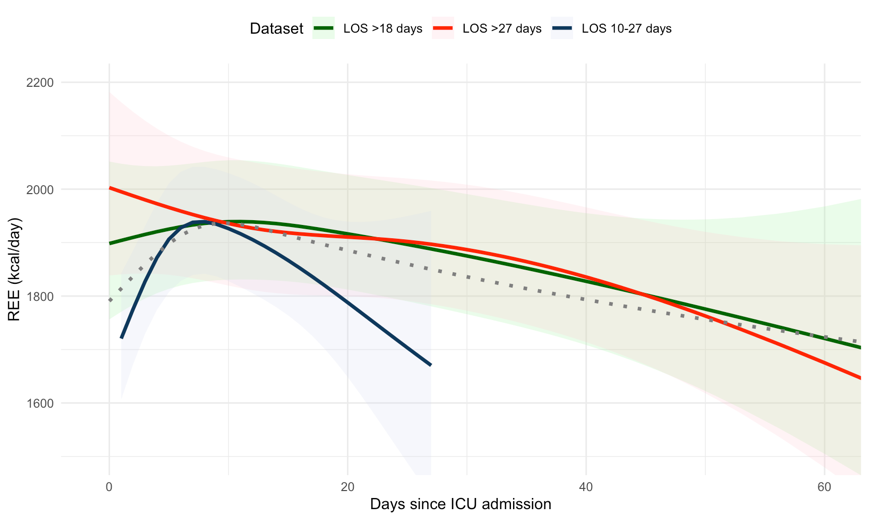

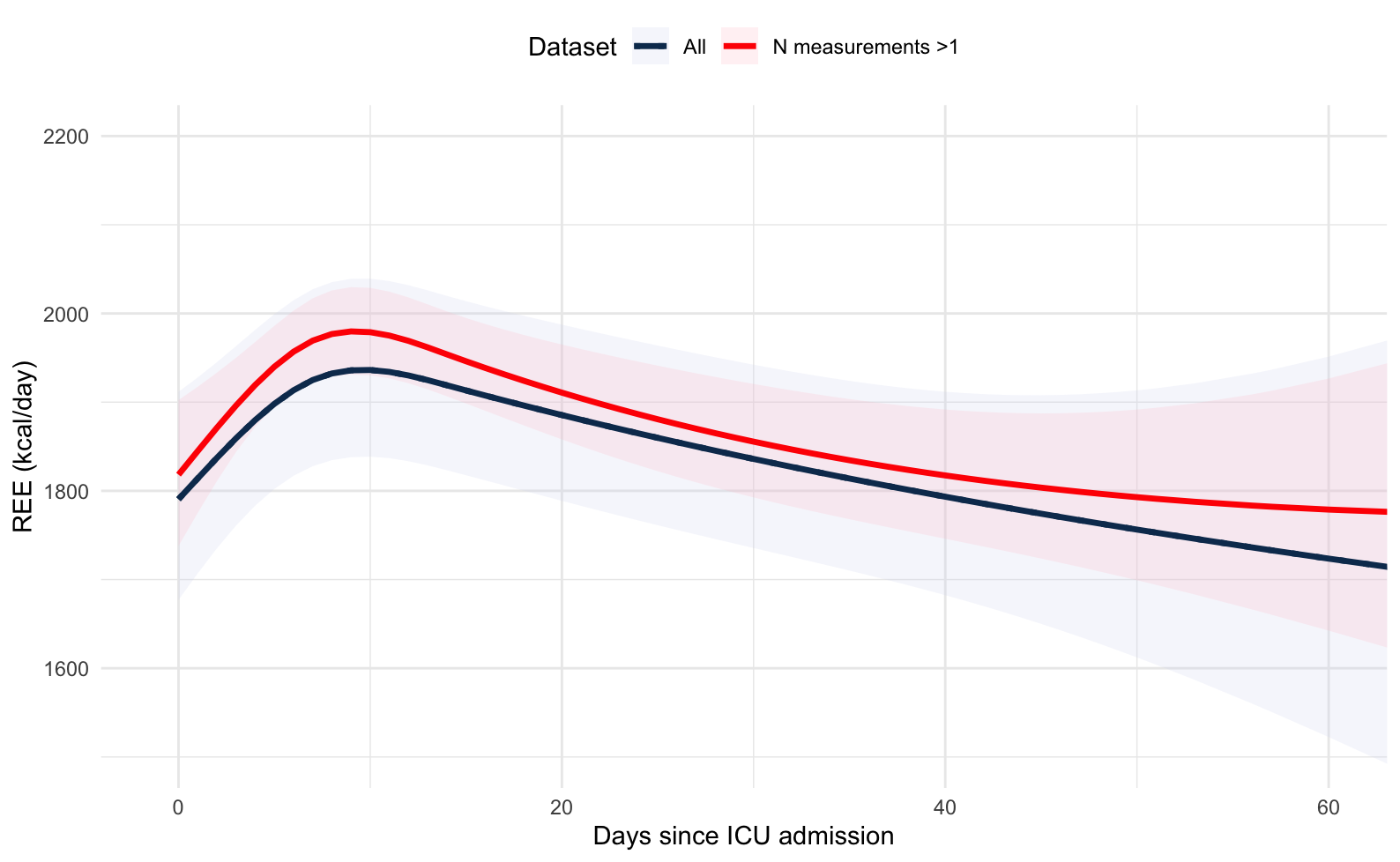


**A**

**B**

**C**

**Supplemental Table 10 – Subanalysis LOS >28 days**

As a subanalysis, we explored whether patients with the most extended ICU stay (n = 96, top quartile) differed in demographic or illness-specific factors (Suppl. Table 10 ). This subgroup did not differ in terms of age, sex, body composition, diagnostic classes, or illness severity scores. ICU mortality was similar. On measurement days, patients used fewer vasopressors, underwent less renal replacement therapy, and required less sedation. CRP, albumin, and haemoglobin were lower, whereas urea and UCR were higher. Energy expenditure was similar, but the respiratory quotient was slightly higher beyond 28 days. More energy was delivered (94% vs 86%, p < 0.001).

|  | | **Included pat vs top quartile LOS** | | | | | |
| --- | --- | --- | --- | --- | --- | --- | --- |
|  | | **>28 days**  N = 96*^1^* | | **10-27 days**  N = 292*^1^* | | **p-value** | |
| **Patient Characteristics** | | | | | | | |
| Age (years) | | 56 (16) | | 56 (16) | | 0.7 | |
| Male | | 64 (67%) | | 206 (71%) | | 0.5 | |
| Height (cm) | | 173 (10) | | 173 (10) | | >0.9 | |
| Weight (kg) | | 88 (23) | | 87 (25) | | 0.9 | |
| BMI (kg/m^2^) | | 29 (7) | | 29 (8) | | 0.9 | |
| Admission source | |  | |  | | 0.4 | |
| Emergency department | | 29 (30%) | | 116 (40%) | |  | |
| Ward | | 32 (33%) | | 77 (26%) | |  | |
| Operating theatre | | 23 (24%) | | 63 (22%) | |  | |
| Other ICU | | 12 (13%) | | 36 (12%) | |  | |
| Surgery prior to admission | | 40 (42%) | | 91 (31%) | | 0.059 | |
| Emergency surgery | | 29 (73%), n = 40 | | 74 (81%), n = 91 | | 0.3 | |
| Number of comorbidities | | 1.94 (1.14) | | 2.01 (1.08) | | 0.6 | |
| **Severity** | | | | | | | |
| SOFA score at admission | | 8.4 (3.6), n = 49 | | 8.1 (3.7), n = 147 | | 0.5 | |
| APACHE II score | | 20 (9), n = 47 | | 19 (7), n = 125 | | 0.7 | |
| APACHE IV score | | 115 (38), n = 5 | | 78 (23), n = 11 | | 0.069 | |
| SAPS III score | | 61 (16), n = 32 | | 59 (17), n = 126 | | 0.4 | |
| SAPS II score | | 57 (16), n = 15 | | 54 (16), n = 39 | | 0.5 | |
| Predicted mortality | | 0.29 (0.25) | | 0.26 (0.22) | | 0.4 | |
| No prediction score | | 2 (2.1%) | | 2 (0.7%) | | 0.3 | |
| **ICU details** | | | | | | | |
| Vasopressor during ≥1 measurement | | 77 (80%) | | 220 (75%) | | 0.3 | |
| Dialysis during ≥1 measurement | | 30 (31%) | | 66 (23%) | | 0.089 | |
| Length of stay, incl. previous ICU | | 39 days (33 days - 49 days) | | 16 days (13 days - 21 days) | | **<0.001** | |
| Sepsis diagnosis during stay | |  | |  | | 0.3 | |
| Sepsis | | 14 (15%) | | 32 (11%) | |  | |
| Septic shock | | 19 (20%) | | 45 (15%) | |  | |
| No sepsis diagnosis | | 63 (66%) | | 215 (74%) | |  | |
| Alive at ICU discharge | | 80 (83%) | | 244 (84%) | | >0.9 | |
| Length of stay | | 19 (13) | | 8 (6) | | **<0.001** | |
|  |  | |  | |  | |  |
|  |  | |  | |  | |  |

|  |  |  |  |  |  |
| --- | --- | --- | --- | --- | --- |
|  | | **Included pat vs LOS >28** | | | |
|  | | **>28 days**  N = 444*^1^* | | **10-27 days**  N = 655*^1^* | **p-value** |
| **Indirect calorimetry** | | | | | |
| Energy expenditure (kcal/day) | | 1,895 (1,569, 2,217) | | 1,849 (1,570, 2,243) | 0.8 |
| Energy expenditure/adjusted body weight (kcal/kg/day) | | 25.4 (21.8, 29.0) | | 25.0 (21.6, 29.3) | >0.9 |
| Respiratory quotient | | 0.82 (0.77, 0.88) | | 0.80 (0.75, 0.86) | **<0.001** |
| VO_2_ | | 277 (226, 321) | | 268 (227, 328) | >0.9 |
| VCO_2_ | | 229 (187, 262) | | 219 (183, 259) | 0.13 |
| **Administered nutrition** | | | | | |
| Enteral nutrition | | 378 (85%) | | 547 (84%) | 0.5 |
| Kcal / day | | 1,542 (719) | | 1,434 (658) | **0.014** |
| Prot / day (g) | | 86 (47) | | 84 (45) | 0.4 |
| Carbohydrates / day (g) | | 152 (84) | | 145 (71) | 0.2 |
| Lipids / day (g) | | 64 (35) | | 57 (27) | **0.004** |
| Parenteral nutrition | | 88 (20%) | | 87 (13%) | **0.004** |
| Kcal / day | | 1,355 (573) | | 1,230 (565) | 0.13 |
| Protein / day (g) | | 75 (35) | | 66 (31) | 0.12 |
| Carbohydrates / day (g) | | 159 (127) | | 135 (69) | 0.13 |
| Lipids / day (g) | | 51 (22) | | 46 (21) | 0.2 |
| Glucose infusion | | 108 (24%) | | 252 (38%) | **<0.001** |
| Kcal from glucose infusions / day | | 167 (267) | | 166 (196) | 0.054 |
| Amino acid infusion | | 35 (7.9%) | | 27 (4.1%) | **0.008** |
| No nutrition | | 15 (3.4%) | | 28 (4.3%) | 0.5 |
| Kcal from propofol / day | | 321 (167) | | 309 (158) | 0.5 |
| Lipids from propofol / day (g) | | 32 (17) | | 31 (16) | 0.5 |
| Total kcal / day | | 1,812 (680) | | 1,666 (682) | **<0.001** |
| Total kcal / day / REE (%) | | 94 (33) | | 86 (34) | **<0.001** |
| **SOFA, CRRT, fever** | | | | | |
| SOFA score at measurement | | 6 (4, 8), n = 263 | | 7 (4, 9), n = 354 | 0.050 |
| Temperature >38.5°C within 2 h | | 38 (8.6%) | | 59 (9.0%) | 0.8 |
| Continuous renal replacement therapy | | 96 (22%) | | 117 (18%) | 0.12 |
| **Chemistry** | | | | | |
| Hemoglobin (g/L) | | 86 (14) | | 93 (17) | **<0.001** |
| CRP (mcmol/L) | | 120 (98) | | 134 (109) | 0.094 |
| Albumin (g/L) | | 21.7 (5.4) | | 24.3 (5.8) | **<0.001** |
| Urea (mcmol/L) | | 14 (9) | | 12 (9) | **<0.001** |
| Creatinine (mcmol/L) | | 96 (75) | | 108 (97) | 0.10 |
| Urea:creatinine ratio | | 184 (236) | | 148 (275) | **<0.001** |
| **Ventilation and circulation** | | | | | |
| Invasive ventilation | | 443 (100%) | | 655 (100%) | 0.4 |
| FiO_2_ | | 0.34 (0.10) | | 0.32 (0.10) | 0.088 |
| PEEP (cmH_2_O) | | 8.3 (2.4) | | 8.6 (2.3) | 0.083 |
| Noradrenaline | | 187 (42%) | | 328 (50%) | **0.009** |
| No vasopressor | | 245 (55%) | | 309 (47%) | **0.009** |
| **Analgesia and sedation** | | | | | |
| RASS | |  | |  | **<0.001** |
| Deeply sedated (-5 - -3) | | 193 (44%) | | 408 (63%) |  |
| Mildly sedated (-2 - 0) | | 226 (51%) | | 212 (33%) |  |
| Agitated (+1 - +4) | | 23 (5.2%) | | 31 (4.8%) |  |
| Propofol dose (mg/kg/h) | | 1.78 (0.90), n = 160 (36%) | | 1.70 (0.82), n = 405 (62%) | 0.5 |
| Dexmedetomidine | | 54 (12%) | | 64 (9.8%) | 0.2 |
| Thiopenthone | | 21 (4.7%) | | 9 (1.4%) | **<0.001** |
| Ketamine | | 18 (4.1%) | | 26 (4.0%) | >0.9 |
| Clonidine | | 25 (5.6%) | | 66 (10%) | **0.009** |
| No sedative | | 199 (45%) | | 185 (28%) | **<0.001** |
| Epidural analgesia | | 6 (1.4%) | | 18 (2.7%) | 0.12 |
| Parenteral opioids | | 289 (65%) | | 460 (70%) | 0.073 |
| Paracetamol | | 265 (60%) | | 360 (55%) | 0.12 |
|  |  |  |  |  |  |
|  |  |  |  |  |  |

**Supplemental Table 11**

Model fit diagnostics for the LCA. The adjusted model includes a correlated slope, whereas the other models do not allow correlated slopes. Random effects include only patient ID. BIC = Bayesian information criterion; AIC = Akaike’s information criterion; Loglik = log-likelihood; df = degree of freedom

| **Model** | **BIC** | **AIC** | **Loglik** | **Entropy** | **Class 1 (%)** | **Class 2 (%)** | **Class 3 (%)** | **Class 4 (%)** | **Class 5 (%)** | **Class 6 (%)** |
| --- | --- | --- | --- | --- | --- | --- | --- | --- | --- | --- |
| Adjusted, no corr slope, 1 df | -523 | -595 | 317 | 1.00 | 100 |  |  |  |  |  |
| Adjusted, 1 df | -531 | -610 | 327 | 1.00 | 100 |  |  |  |  |  |
| Adjusted, no corr slope, 2 df | -512 | -602 | 326 | 0.26 | 28 | 72 |  |  |  |  |
| Adjusted, 2 df | -510 | -607 | 331 | 0.18 | 32 | 68 |  |  |  |  |
| **Adjusted, no corr slope, 3 df** | **-507** | **-614** | **337** | **0.63** | **11** | **4** | **85** |  |  |  |
| Adjusted, 3 df | -497 | -612 | 338 | 0.45 | 4 | 26 | 70 |  |  |  |
| Adjusted, no corr slope, 4 df | -479 | -605 | 338 | 0.40 | 12 | 32 | 52 | 4 |  |  |
| Adjusted, 4 df | -475 | -608 | 341 | 0.55 | 19 | 6 | 2 | 73 |  |  |
| Adjusted, no corr slope, 5 df | -466 | -610 | 345 | 0.64 | 6 | 32 | 6 | 50 | 6 |  |
| Adjusted, 5 df | -448 | -598 | 341 | 0.37 | 42 | 7 | 48 | 0 | 3 |  |
| Adjusted, no corr slope, 6 df | -435 | -597 | 343 | 0.48 | 7 | 16 | 9 | 30 | 32 | 6 |
| Adjusted, 6 df | -420 | -588 | 341 | 0.30 | 58 | 7 | 32 | 0 | 0 | 3 |

**Supplemental Table 12 & 13 – Demographic and clinical characteristics by LCA class.**

| Suppl. Table 12 | **Overall** N = 269*^1^* | **Hyper**  **1** N = 29*^1^* | **Hypo**  **2** N = 11*^1^* | **Normo**  **3** N = 229*^1^* |
| --- | --- | --- | --- | --- |
| **Patient Characteristics** | | | | |
| Age (years) | 57 (16) | 54 (14) | 51 (15) | 58 (16) |
| Male (n) | 181 (67%) | 16 (55%) | 10 (91%) | 155 (68%) |
| Height (cm) | 173 (10) | 172 (11) | 173 (7) | 173 (10) |
| Weight (kg); adjusted if BMI >25 | 75 (12) | 75 (15) | 71 (11) | 75 (12) |
| BMI (kg/m^2^) | 29 (8) | 30 (7) | 27 (8) | 29 (8) |
| Admission source (n) |  |  |  |  |
| Emergency department | 84 (31%) | 10 (34%) | 2 (18%) | 72 (31%) |
| Ward | 83 (31%) | 12 (41%) | 5 (45%) | 66 (29%) |
| Operating theatre | 57 (21%) | 5 (17%) | 3 (27%) | 49 (21%) |
| Other ICU | 45 (17%) | 2 (6.9%) | 1 (9.1%) | 42 (18%) |
| Surgery prior to admission (n) | 99 (37%) | 7 (24%) | 5 (45%) | 87 (38%) |
| Emergency surgery (n) | 78 (79%) | 7 (100%) | 4 (80%) | 67 (77%) |
| Number of comorbidities (n) |  |  |  |  |
| 0 | 104 (39%) | 14 (48%) | 2 (18%) | 88 (38%) |
| 1 | 86 (32%) | 8 (28%) | 3 (27%) | 75 (33%) |
| 2 | 48 (18%) | 5 (17%) | 4 (36%) | 39 (17%) |
| 3 | 21 (7.8%) | 2 (6.9%) | 1 (9.1%) | 18 (7.9%) |
| 4 | 8 (3.0%) | 0 (0%) | 1 (9.1%) | 7 (3.1%) |
| 5 | 2 (0.7%) | 0 (0%) | 0 (0%) | 2 (0.9%) |
| 6 | 0 (0%) | 0 (0%) | 0 (0%) | 0 (0%) |
| **Severity** | | | | |
| SOFA score at admission | 7.9 (3.6), n = 158 | 7.2 (3.3), n = 15 | 7.8 (5.2), n = 8 | 7.9 (3.5), n = 135 |
| APACHE II score | 20 (8), n = 95 | 16 (9), n = 13 | 14 (4), n = 4 | 21 (8), n = 78 |
| APACHE IV score | 90 (32), n = 16 | 79 (21), n = 3 | 67 (NA), n = 1 | 94 (35), n = 12 |
| SAPS III score | 61 (16), n = 136 | 61 (15), n = 14 | 54 (15), n = 6 | 61 (16), n = 116 |
| SAPS II score | 51 (13), n = 34 | 39 (13), n = 2 | 44 (NA), n = 1 | 52 (13), n = 31 |
| No prediction score (n) | 3 (1.1%) | 0 (0%) | 0 (0%) | 3 (1.3%) |
| Predicted mortality | 0.28 (0.24) | 0.26 (0.23) | 0.16 (0.15) | 0.28 (0.24) |
| **ICU details** | | | | |
| Vasopressor during ≥1 measurement (n) | 218 (81%) | 26 (90%) | 10 (91%) | 182 (79%) |
| Dialysis during ≥1 measurement (n) | 69 (26%) | 12 (41%) | 5 (45%) | 52 (23%) |
| Length of stay, incl. previous ICU | 21 days (15 days - 34 days) | 22 days (17 days - 36 days) | 19 days (15 days - 21 days) | 21 days (15 days - 34 days) |
| Sepsis diagnosis during stay (n) |  |  |  |  |
| Sepsis | 36 (13%) | 5 (17%) | 1 (9.1%) | 30 (13%) |
| Septic shock | 53 (20%) | 9 (31%) | 2 (18%) | 42 (18%) |
| No sepsis diagnosis | 180 (67%) | 15 (52%) | 8 (73%) | 157 (69%) |
| Alive at ICU discharge (n) | 226 (84%) | 24 (83%) | 8 (73%) | 194 (85%) |
| *^1^*Mean (SD); n (%); Mean (SD), n = N Non-missing; n (%), n = N; Median (Q1 - Q3) | | | | |

| **Suppl. Table 13** | **Overall**  N = 269*^1^* | **Hyper**  N = 29*^1^* | **Hypo**  N = 11*^1^* | **Normo**  N = 229*^1^* |
| --- | --- | --- | --- | --- |
| **Indirect calorimetry** |  |  |  |  |
| Number of measurements per patient | 3 (2 – 4) | 4 (3 – 6) | 3 (2.5 – 3.5) | 3 (2 – 3) |
| Day in ICU at measurement number: |  |  |  |  |
| 1^a^ | 4 (2– 7), n = 250 | 3 (2 – 4), n = 25 | 4 (3 – 7), n = 10 | 4 (3 – 8), n = 215 |
| 2 | 9 (6 – 13), n = 263 | 7 (6 – 9), n = 27 | 7 (6 – 11), n = 11 | 9 (6 – 13), n = 225 |
| 3 | 12 (10 –18), n = 162 | 11 (10 – 15), n = 22 | 12 (10 – 14), n = 8 | 13 (10 – 18), n = 132 |
| Day in ICU at measurement (median) | 4 (3 - 8) | 3 (2 - 7) | 4 (3 - 9) | 5 (3 - 8) |
| Resting energy expenditure (kcal/day) | 1,807 (1,472 - 2,164) | 2,458 (2,112 - 2,772) | 1,063 (935 - 1,271) | 1,788 (1,476 - 2,005) |
| Resting energy expenditure/adjusted body weight (kcal/kg/day) | 23.8 (20.6 - 28.7) | 31.9 (29.6 - 34.3) | 15.2 (12.7 - 17.3) | 23.2 (20.6 - 27.9) |
| Respiratory quotient | 0.80 (0.74 - 0.86) | 0.77 (0.70 - 0.83) | 0.79 (0.70 - 0.86) | 0.80 (0.75 - 0.87) |
| VO_2_ | 261 (213 - 313) | 366 (300 - 397) | 154 (136 - 187) | 259 (214 - 296) |
| VCO_2_ | 208 (173 - 248) | 267 (225 - 319) | 126 (108 - 152) | 205 (176 - 246) |
| Administered nutrition |  |  |  |  |
| Enteral nutrition | 207 (77%) | 20 (69%) | 9 (82%) | 178 (78%) |
| Kcal / day | 1,200 (576 - 1,663) | 896 (468 - 1,611) | 755 (720 - 1,289) | 1,210 (605 - 1,680) |
| Prot / day (g) | 63 (30 - 96) | 51 (24 - 90) | 45 (36 - 58) | 67 (33 - 96) |
| Lipids / day (g) | 43 (24 - 66) | 30 (18 - 60) | 31 (25 - 59) | 47 (24 - 67) |
| Carbohydrates / day (g) | 119 (57 - 170) | 97 (51 - 179) | 82 (67 - 106) | 125 (57 - 170) |
| Parenteral nutrition | 32 (12%) | 1 (3.4%) | 2 (18%) | 29 (13%) |
| Protein / day (g) | 61 (35 - 87) | 82 (82 - 82) | 78 (30 - 125) | 59 (35 - 87) |
| Lipids / day (g) | 41 (25 - 61) | 38 (38 - 38) | 21 (21 - 21) | 43 (27 - 61) |
| Carbohydrates / day (g) | 112 (83 - 167) | 79 (79 - 79) | 148 (58 - 238) | 114 (90 - 167) |
| Kcal / day | 1,104 (755 - 1,628) | 1,026 (1,026 - 1,026) | 948 (565 - 1,331) | 1,104 (770 - 1,628) |
| Glucose infusion | 130 (48%) | 19 (66%) | 6 (55%) | 105 (46%) |
| Kcal from glucose infusions / day | 202 (48 - 403) | 202 (48 - 403) | 206 (202 - 403) | 202 (48 - 384) |
| Amino acid infusion | 19 (7.1%) | 2 (6.9%) | 1 (9.1%) | 16 (7.0%) |
| No nutrition | 12 (4.5%) | 3 (10%) | 0 (0%) | 9 (3.9%) |
| Kcal from propofol / day | 288 (192 - 432) | 242 (180 - 432) | 258 (229 - 480) | 300 (192 - 420) |
| Lipids from propofol / day (g) | 29 (19 - 43) | 24 (18 - 43) | 26 (23 - 48) | 30 (19 - 42) |
| Total kcal / day | 1,440 (940 - 1,909) | 1,152 (643 - 1,844) | 1,500 (1,166 - 1,811) | 1,445 (943 - 1,949) |
| Total kcal / day / REE (%) | 78 (53 - 104) | 57 (35 - 73) | 146 (92 - 170) | 81 (55 - 104) |
| **Organ support** |  |  |  |  |
| Invasive ventilation | 269 (100%) | 29 (100%) | 11 (100%) | 229 (100%) |
| FiO_2_ | 0.30 (0.25 - 0.40) | 0.40 (0.30 - 0.40) | 0.25 (0.25 - 0.30) | 0.30 (0.25 - 0.40) |
| PEEP | 8.0 (7.0 - 10.0) | 10.0 (8.0 - 10.0) | 8.0 (6.0 - 10.0) | 8.0 (7.0 - 10.0) |
| SOFA score at measurement | 8 (6 - 10), n = 171 | 9 (6 - 12), n = 14 | 9 (7 - 14), n = 7 | 8 (5 - 10), n = 150 |
| Noradrenaline | 182 (68%) | 23 (79%) | 8 (73%) | 151 (66%) |
| No vasopressor | 82 (30%) | 6 (21%) | 2 (18%) | 74 (32%) |
| Continuous renal replacement therapy | 52 (19%) | 8 (28%) | 4 (36%) | 40 (17%) |
| Chemistry |  |  |  |  |
| Hemoglobin (g/L) | 91 (79 - 107) | 98 (83 - 111) | 98 (81 - 105) | 90 (79 - 107) |
| CRP (mcmol/L) | 137 (61 - 238) | 172 (60 - 333) | 93 (68 - 179) | 140 (61 - 229) |
| Albumin (g/L) | 24.0 (19.0 - 28.0) | 22.0 (17.0 - 27.0) | 24.0 (23.0 - 29.0) | 24.0 (20.0 - 27.0) |
| Urea (mcmol/L) | 10 (6 - 16) | 12 (9 - 16) | 10 (4 - 24) | 10 (6 - 16) |
| Creatinine (mcmol/L) | 85 (59 - 135) | 114 (65 - 176) | 130 (61 - 206) | 82 (59 - 125) |
| Urea:creatinine ratio | 108 (74 - 151) | 106 (80 - 174) | 82 (53 - 117) | 109 (75 - 149) |
| **Analgesia and sedation** |  |  |  |  |
| Temperature >38.5°C within 2 h | 18 (6.7%) | 3 (10%) | 0 (0%) | 15 (6.6%) |
| RASS |  |  |  |  |
| Deeply sedated (-5 - -3) | 192 (71%) | 24 (83%) | 6 (55%) | 162 (71%) |
| Mildly sedated (-2 - 0) | 72 (27%) | 5 (17%) | 5 (45%) | 62 (27%) |
| Agitated (+1 - +4) | 5 (1.9%) | 0 (0%) | 0 (0%) | 5 (2.2%) |
| Propofol dose (mg/kg/h) | 2.65 (1.71 - 3.37), n = 189 (70%) | 2.31 (1.74 - 2.83), n = 22 (76%) | 2.64 (1.90 - 3.28), n = 8 (73%) | 2.68 (1.65 - 3.56), n = 159 (69%) |
| Dexmedetomidine | 20 (7.4%) | 1 (3.4%) | 0 (0%) | 19 (8.3%) |
| Thiopenthone | 5 (1.9%) | 1 (3.4%) | 0 (0%) | 4 (1.7%) |
| Ketamine | 9 (3.3%) | 0 (0%) | 1 (9.1%) | 8 (3.5%) |
| Clonidine | 32 (12%) | 4 (14%) | 1 (9.1%) | 27 (12%) |
| No sedative | 45 (17%) | 2 (6.9%) | 3 (27%) | 40 (17%) |
| Epidural analgesia | 6 (2.2%) | 0 (0%) | 0 (0%) | 6 (2.6%) |
| Parenteral opioids | 200 (74%) | 23 (79%) | 9 (82%) | 168 (73%) |
| Paracetamol | 142 (53%) | 17 (59%) | 2 (18%) | 123 (54%) |
| ^a^Might differ from the total number of patients, as no missing data in any of the covariates was allowed. | | | | |

**References**

1. Jonckheer J, Spapen H, Malbrain M, Oschima T, De Waele E, (2020) Energy expenditure and caloric targets during continuous renal replacement therapy under regional citrate anticoagulation. A viewpoint. Clin Nutr 39: 353-357

2. Achamrah N, Delsoglio M, De Waele E, Berger MM, Pichard C, (2021) Indirect calorimetry: The 6 main issues. Clin Nutr 40: 4-14
